# Radon-Guided Wavelet-Domain Attention U-Net for Periodic Artifact Suppression in Brain MRI

**DOI:** 10.3390/jimaging12040153

**Published:** 2026-04-02

**Authors:** Jesus David Rios-Perez, German Sanchez-Torres, John W. Branch-Bedoya, Camilo Andres Laiton-Bonadiez

**Affiliations:** 1 Departamento de Ciencias de la Computación y de la Decisión, Facultad de Minas, Universidad Nacional de Colombia, Medellín 050035, Colombia; jwbranch@unal.edu.co (J.W.B.-B.); claiton@unal.edu.co (C.A.L.-B.); 2 Facultad de Ingeniería, Grupo de Investigación y Desarrollo en Sistemas y Computación, Universidad del Magdalena, Santa Marta 470004, Colombia; gsanchez@unimagdalena.edu.co

**Keywords:** MRI, artifact correction, self-attention, wavelet, U-Net

## Abstract

Periodic artifacts such as ringing (Gibbs), herringbone (spike/corduroy), and zipper patterns degrade the quality of brain MRI. We present a reproducible framework that (i) synthetically generates periodic artifacts with controllable severity directly in k-space, (ii) normalizes pattern orientation through a Radon-guided alignment step, and (iii) corrects them in the wavelet domain using a 2D DWT (AA/AD/DA/DD) with a band-weighted loss. The evaluation was conducted using DLBS T1-weighted 3T MRI volumes with synthetically generated periodic artifacts. It combined global image-quality metrics (SSIM, PSNR) with per-band metrics to quantify how correction concentrates on high-frequency components, and included ablation studies, mixed-artifact stress tests, and structural preservation analyses. Compared with several baseline architectures, the proposed approach shows improvements in structural fidelity and a reduction in periodic patterns (SSIM: 0.985±0.022; PSNR: 43.337±5.364; reduction in concentrated error in high-frequency bands), while preserving unaffected structures. These findings indicate that, within a controlled synthetic benchmark, aligning the pattern orientation prior to learning and optimizing correction in the wavelet domain enables suppression of synthetically generated periodic artifacts while limiting over-smoothing.

## 1. Introduction

Magnetic resonance imaging (MRI) is a widely used non-invasive tool in clinical diagnosis and biomedical research, due to its ability to enhance soft-tissue contrast and versatility in anatomical, functional, and metabolic applications. Millions of MRI scans are performed worldwide each year. However, the increasing demand for higher spatial and temporal resolution also increases exposure to artifacts that degrade anatomical and quantitative fidelity, which may affect early diagnosis and follow-up accuracy. These artifacts fall into three main categories: (1) high noise and limitations in spatial resolution; (2) geometric distortions caused by magnetic field heterogeneities and patient motion; and (3) susceptibility artifacts associated with fast gradient echo (FGE) sequences or the presence of metallic structures [1,2].

In multi-shot diffusion-weighted imaging (DWI), for example, lengthening the acquisition time to optimize the signal-to-noise ratio (SNR) increases vulnerability to distortions and motion shifts [3]. Similarly, in whole-body positron emission tomography (PET)/MRI studies, metallic implants and truncation of the field of view cause areas of signal loss that hinder attenuation correction and tracer uptake quantification [4]. Furthermore, echo planar imaging (EPI) sequences used in functional neuroimaging show more pronounced spatial aberrations at ≥7 T [2]. In oncology applications and cardiac balanced steady-state free precession (bSSFP) cine imaging, noise, distortions, banding, and flow artifacts hinder the detection of small lesions and morphological and functional analysis [5]. Given this complex interaction of protocols and artifacts, artificial intelligence (AI)-based post-processing strategies are required to restore image quality and ensure the reproducibility of quantitative measurements in diverse clinical scenarios.

In recent years, deep learning has become a widely adopted strategy for correcting these artifacts in MRI. Deformable convolutional networks (DCNs) have been reported to be effective in correcting geometric distortions in neuroimaging while also extracting features relevant for tumor classification [6]. In multi-shot DWI, denoising schemes trained on single-shot data have allowed acquisitions to be accelerated up to 4×, maintaining fidelity at high b-values, and improving the detectability of rectal lesions [3]. For PET/MRI, deep completion methods estimate signal-loss regions caused by metal artifacts or truncation, reducing the volumetric error in attenuation correction from 9.8% to less than 1% in the head and torso [4].

In cardiac bSSFP cine imaging, dual-encoder architectures have jointly suppressed banding and flow artifacts, outperforming conventional averaging [5], while the TS-Net network combines inverted phase encodings (PEs) with anatomical images to correct distortions in all three directions without requiring additional data during inference [2]. Despite these quantitative improvements, most proposals are evaluated in specific domains using particular protocols, lack direct comparisons with traditional techniques, and offer limited validation for explainability, generalizability, and standardized clinical metrics [1]. This fragmentation makes it difficult to identify the most robust approaches and to implement them in heterogeneous environments.

In this work, we propose a reproducible synthetic framework for generating periodic artifacts (ringing, herringbone, and zipper) in reference structural images, with k-space severity control. The protocols parameterize smoothing, axis/direction, kernel size, distance, and amplitude, among other parameters, with calibrated ranges to obtain controlled variations in artifact severity. This design enables the study of how correction methods behave under increasingly severe regimes.

At the learning stage, we introduce Radon-guided orientation normalization. We estimate the dominant pattern angle and apply a noisy and ground truth (GT) rotation before the network, then reverse the rotation during reconstruction, thereby reducing geometric variability that hinders training. The core model, WaveletBasedAttention-Net, uses a 2D discrete wavelet transform (DWT) to decompose each slice into four bands [Approximation + Approximation (AA), Approximation + Detail (AD), Detail + Approximation (DA), and Detail + Detail (DD)]. The resulting four-channel tensor is processed by a U-Net architecture with four-channel input and output, employing attention-based skip connections that enhance feature selection and spatial representation. The corrected bands are then reconstructed using an inverse discrete wavelet transform (IDWT). The WaveletLoss function weights the error per band to selectively suppress the pattern, and we record the structural similarity index measure (SSIM) during training and inference as a global structural metric.

In the Section 4, we evaluate the model on a controlled synthetic dataset using global and wavelet-domain metrics, along with partitioning analyses, ablation studies, structural preservation assessment, and mixed-artifact stress tests. Regarding the data partitioning analysis, while histograms and PCA projections indicate substantial overlap between the training and validation subsets, formal two-sample tests detect subtle but statistically significant differences. Therefore, the experimental setting is best understood as a controlled synthetic evaluation rather than a statistically equivalent sampling regime.

## 2. Previous Works

### 2.1. Sampling/Aliasing/Truncation-Related Artifacts

This category encompasses the effects that arise when the signal is discretized in space and frequency. These include aliasing due to undersampling, Gibbs ringing caused by truncation in k-space, and wrap-around, where a signal from outside the field of view is folded onto the image. To mitigate these effects, accelerated sampling techniques (parallel imaging, compressed sensing, and simultaneous multislice sampling) are now combined with transform-domain methods and spatiotemporal regularization. Furthermore, approaches that model the acquisition physics and integrate neural networks (in k-space, in the image domain, or in both domains) are gaining ground, thereby restoring the signal and suppressing artifacts without always relying on fully sampled references.

For noise suppression and defocusing, the goal is to eliminate interference and recover fine detail in dynamic sequences. In the k-t domain, models such as the DENSE Artifact Suppression Network (DAS-Net) separate echoes in dense cine, and deep residual networks preserve edges in accelerated multi-shot diffusion [1,2]. In more “real-world” scenarios, vendor-agnostic convolutional neural network (CNN) filtering applied to DICOM images increased SSIM while saving time. In addition, a 2D U-Net trained with simulated degradations (Gaussian noise, blur, and motion) clearly outperformed classical methods in denoising and deblurring [3,4].

In arterial spin labeling (ASL) perfusion, deep autoencoders improve SNR and reduce kinetic error, supporting their use for quantitative maps such as cerebral blood flow (CBF) [5]. In parallel, zero-shot in situ schemes (e.g., zero-shot medical image artifact reduction (ZSAR)/one-shot medical image artifact reduction (OSAR)) reduce dependence on ground truth, although they degrade more at low SNR, in 3D settings, and when manual regions of interest (ROI) are required [6,7]. For mixed artifacts, dual-domain architectures such as feature distillation block (FDB-GAN) (high-frequency k-space branch + spatial branch) achieve net advantages, albeit with greater complexity and limited 3D validation, and low-SNR evaluation [8].

In clinical and commercial settings, deep learning reconstruction (DLR) in 3D T2W-FS increases SNR and decreases ringing without prolonging acquisition, improving the area under the curve (AUC) of findings (e.g., epidural fluid) [9]. In hybrid super-resolution, orthogonal stacks plus a 5-layer artifact reduction convolutional neural network (ARCNN) achieve competitive PSNR and SSIM, albeit with high computational costs and limited pathological validation [10]. For out-of-site generalization, a residual network (ResNet) with perceptual loss improves PSNR, SSIM, and normalized root mean square error (NRMSE) without retraining but may attenuate subtle hyperintensities; further validation in 3D and other pathologies is suggested [11]. In Rician noise, patched residual frames recover fine texture, and residual noise learning approaches show improvements in PSNR and SSIM, although sometimes with phantom-based evidence and without 3D clinical validation [12,13]. In specific contexts, AIR™ Recon Deep Learning (DL) for prostate imaging (residual encoder trained with “near-perfect” vs. conventional pairs) obtained a higher frequency of “excellent” ratings and a reduction of artifacts, improving anatomical visualization. However, objective metrics and slice-by-slice correlation with pathology were not reported [14]. In cardiac late gadolinium enhancement (LGE), a deep learning reconstruction (DLR) prototype increased SNR (up to ~3× in phantom and ~1.8–3× in clinical) and sharpness, but high levels of noise reduction altered quantitative measurements with conventional thresholds; the full width at half maximum (FWHM) method proved more stable, underscoring the need for systematic evaluations of quantification [15].

When k-space lines are missing, aliasing occurs. To strengthen generalized auto-calibrating partially parallel acquisitions (GRAPPAs) without external data, scan-specific artifact reduction in k-space (SPARK) learns to correct the error in the auto-calibration signal (ACS) region; the alias artifact suppression network (ALIASNET) reduces parameters by combining 1D regularizers with 2D convolutions [16,17]. In real-time cine imaging without ground truth, a CNN is pre-trained with synthetic profiles and subsequently refined with data consistency [18]. Several studies reveal that 2D spatiotemporal U-Nets match 3D approaches with less data (supported by persistent homology), and that implicit approaches such as neural implicit k-space (NIK), sinusoidal multi-layer perceptron (MLP), or multiresolution deformable convolutions improve end-to-end quality [19,20,21]. The k-space/image jointly unrolled cross-domain optimization-based spatiotemporal reconstruction network (JUST-Net) improves myelin maps and mitigates motion; and recurrent frames or framework like Flow Reconstruction and Segmentation for Low-Latency Cardiac Output (FReSCO) allow inference in <1 s, which is useful for real-time applications [22,23,24]. In simultaneous multislice (SMS) myocardial perfusion, both signal intensity informed multi-coil (SIIM) and edge-guided cascades enforce data/space consistencies to stabilize the reconstruction [25,26].

In dual-domain architectures, multi-domain convolutional neural network (MD-CNN) and DuDoRNet+ combine 3D subnets, 2D U-Nets, and interpolation; in plug-and-play magnetic resonance fingerprinting (MRF), the alternating direction method of multipliers (ADMM) integrates a pre-trained denoiser without retraining it; and Regularization by Artifact-REmoval (RARE) extends Regularization by Denoising (RED) with Artifact2Artifact pairs for 4D free-breathing [27,28,29,30]. Other frameworks explore adversarial and transformer-based strategies, including the Hierarchical Perception Adversarial Learning Framework (HP-ALF), a volumetric radial generative adversarial network (GAN), and accelerated MRI reconstruction using a recurrent transformer (ReconFormer). Additional approaches include structured low-rank methods, and task-specific accelerations such as spiral phase-contrast cardiac magnetic resonance imaging (PCMR), ~59 s → 3.9 s [31,32,33,34,35].

In musculoskeletal imaging, Controlled Aliasing in Parallel Imaging Results in Higher Acceleration (CAIPIRINHA) combined with compressed sensing (CS) accelerates 3D Turbo Spin-Echo (TSE) (4–8×), although prospective validations are needed. In prostate multiparametric magnetic resonance imaging (mpMRI)—Prostate Imaging-Reporting and Data System (PI-RADS)—synthetic data and networks without full reference are combined. Variants such as dual GAN-U-Net (DLGAN) or a Hybrid Image-Wavelet Domain Network (HIWDNet), together with modules such as the Cross-scale Dense Feature Fusion Module (CDFFM), Region Adaptive Artifact Removal Module (RAARM), and Wavelet Sub-band Reconstruction Module (WSRM), are also emerging, showing good metrics but with stability and latency compromises [36,37,38,39].

Uncertainty-guided progressive GANs have also been proposed; these refine regions with high uncertainty and improve interpretability with limited data, albeit with greater complexity [40].

### 2.2. Motion Artifacts

Motion artifacts in MRI—blurring, ghosting, or phase inconsistencies—arise from voluntary or involuntary patient movement. Two main families of methods can be used to mitigate them: (i) prospective corrections, which measure movement (e.g., with navigators or sensors) and adjust the acquisition sequence in real time; and (ii) retrospective corrections, which act after acquisition (in k-space or image) using registration, transform inversion, and constraints such as compressed sensing. More recently, deep learning approaches—supervised and unsupervised, using CNNs, GANs, diffusion models, and transforms—learn to “translate” artifacted images into clean images by combining spatial and frequency information.

Non-rigid motion and artifact representation. Non-rigidity motion requires models that adapt to local, anatomy-dependent deformations [41]. In this context, deformable convolutions adaptively shift convolutional kernels (${\Delta p}_{n}$) to follow the underlying geometry and are combined with deformable max pooling and a final Support Vector Machine (SVM) for tumor detection [41]. In parallel, disentangled learning approaches separate structural content from artifact components to better align with Spatial Transformer Networks (STNs) and cross-stitch in nnUNet, optimizing mean absolute error (MAE) and multi-scale structural similarity (MS-SSIM) [42].

Respiratory motion in the abdomen: from filters to U-Nets. In liver MRI, post-processing filters such as Motion Artifact Reduction with Convolutional Neural Network (MARC), shallow CNN, and U-Nets enhanced with high-pass filtering reduce respiratory artifacts and improve SSIM and phase-insensitive contrast [43,44]. In more complex scenarios, generative methods have also been used. In fetal MRI, residual GANs with Squeeze-and-Excitation combine adversarial, L1, and perceptual losses, including the Visual Geometry Group (VGG) perceptual loss [45]. Conditioned diffusion models achieve high SSIM in simulated cardiac cine imaging, while Variance Exploding Stochastic Differential Equation (VE-SDE) models formulate the process in terms of forward and inverse stochastic differential equations (SDEs) with iterative steps in k-space (powerful but computationally costly and with the risk of retaining low-frequency artifacts) [46,47]. Recurrent GANs with multi-scale Convolutional Long-Short Term Memory (ConvLSTM) also improve PSNR and SSIM in temporal interpolation in cine imaging [48], while classic supervised schemes such as U-Net, variational autoencoder (VAE), and GAN remain effective when adequate training references are available [49].

When clean–corrupt pairs are unavailable, unpaired sampling with bootstrap aggregation (averaging several reconstructions) helps discard outliers and outperforms purely supervised alternatives in mono- and multi-coil TSM acquisitions [50]. Furthermore, integrated frameworks that detect, correct, and segment artifacts within a single processing pipeline—such as CNN/Convolutional Recurrent Neural Network (CRNN)/U-Net architectures—show improvements in accuracy and resolution in population datasets such as the UK Biobank [51,52]. A recent review (2018–present) covers approaches ranging from classical coding methods to deep learning, reporting high accuracies (∼97%) and indicating the need for multicenter validation and real-time 3D/4D optimization [53].

Rigid motion: an active line strategy simulates rigid artifacts by replacing k-space lines using temporal masks and trains models to reverse them. For example, Motion Artifact Reduction using a Conditional Diffusion Probabilistic Model (MAR-CDPM) matches or outperforms U-Net, CycleGAN, and Pix2Pix in silico and in multicenter environments [54]. Motion Artifact Unsupervised Disentanglement Generative Adversarial Network (MAUDGAN) achieves competitive results without paired data or sequence modifications, combining adversarial, reconstruction, and artifact losses [55]. Using a hybrid approach, a hybrid Deep AutoEncoder–Convolutional Neural Network (DAE-CNN) first classifies ceT1 volumes as clean or artifacted and then removes artifacts using optimized guided bilateral filtering, achieving high PSNR and classification accuracy; however, 3D validation and detailed analysis in pathological regions are still lacking [56].

### 2.3. Off-Resonance and Susceptibility (B0)

This category encompasses effects that arise when the main magnetic field is not perfectly homogeneous. Typical causes include incomplete shimming, differences in susceptibility between tissues, or the presence of metal. The resulting artifacts are typically observed as geometric distortions, signal mismatches, and off-resonance frequency shifts [57]. To address these effects, the literature generally combines three lines of research: (i) acquisition strategies, (e.g., Slice Encoding for Metal Artifact Correction (SEMAC), View-Angle Tilting (VAT), Multi-Acquisition with Variable Resonance Image Combination (MAVRIC), EPI with reverse polarities, Dixon, and bSSFP with phase-cycling); (ii) physical correction and inversion algorithms; and (iii) deep learning approaches—both supervised and unsupervised—that learn to suppress or compensate for these artifacts [57].

Metal Artifact Reduction Sequence MRI (MARS-MRI): areas of attenuated or absent signal can appear around metallic implants, complicating, for example, the generation of attenuation maps in PET/MRI and potentially inducing significant quantitative biases. One representative approach uses dilated convolutional networks with residual connections to “fill in” the truncated regions by simulating metallic voids in healthy data and optimizing a reconstruction loss based on root mean squared error (RMSE). In tests, artifact volume was substantially reduced, and quantitative biases in the head and thorax were markedly decreased; however, challenges remain, including multicenter validation, data expansion, and coverage of complex implants [58].

In parallel, hardware improvements (e.g., dense radiofrequency (RF) coils, parallel imaging, and gradient defect correction) and dedicated sequences (VAT, SEMAC)—often combined with iterative reconstruction methods or neural networks—help mitigate signal loss, distortion, and frequency shifts. Careful parameter balancing is essential. Increasing bandwidth reduces in-plane distortion but also decreases SNR, while shortening the spin-echo time (TE) refocuses intravoxel dephasing at the cost of a higher specific absorption rate (SAR). In fat suppression, Dixon and Short-Tau Inversion Recovery (STIR) exhibit different sensitivities to B0/B1 inhomogeneities, so the protocol must be tailored to the implant, clinical objective, and time/SAR constraints [59].

Unsupervised learning and multimodal approaches. Unsupervised methods have been proposed to correct off-resonance distortions from dual-polarity EPI acquisitions by estimating a direct field map from both polarities without requiring a distortion-free reference. These approaches show reduced overfitting and good performance under low SNR conditions, although they increase acquisition time (e.g., by requiring both polarities), and further testing is needed across a broader range of implants. In parallel, multimodal architectures for computed tomography (CT)/MRI artifact reduction have been explored that introduce similarity terms between modalities within composite loss functions, with quantitative improvements in CT noise reduction and segmentation propagation in MRI. However, the gains depend on the anatomical region evaluated and the size of the test set, and challenges related to multicenter generalization remain [57,60].

EPI distortion correction. The goal is to estimate a displacement map that reverses geometric deformations and intensity distortions. Established methods, such as TOPUP, estimate the field using regularization, while U-Net-type networks—with losses enforcing cycle consistency, smoothness, or anatomy-based constraints—have accelerated the process and increased structural fidelity [3,61,62].

DLRPG-net restricts the ΔB0 map to a smooth subspace (splines) and simultaneously corrects geometry and intensity, yielding PSNR and SSIM improvements at 3 T and 7 T with very low inference times; however, it requires UP/DOWN pairs, and its robustness to motion and varied protocols requires further study [63].

PreQual synthesizes a “distortion-free” b0 from T1 and a “real” b0 using a 3D GAN. This facilitates subsequent correction with TOPUP but can alter derived metrics (e.g., fractional anisotropy (FA) in tract-based spatial statistics (TBSS)) if applied without reverse-polarity data [62].

S-Net estimates a displacement field from two EPIs with inverted PEs and integrates a differentiable spatial transformer unit, achieving accuracy comparable to TOPUP with substantial computational speedups [64].

TS-Net extends this approach to 3D and incorporates a T1-based anatomical term during training. It outperforms TOPUP and related methods across several datasets (fMRI/DWI, 3T, and 7T) with sub-second graphics processing unit (GPU) inference. Its adoption, however, depends on substantial training, careful hyperparameter selection, and the availability of inverse PE pairs [65].

### 2.4. Ghosting, Phase Errors, and System Effects

This category encompasses artifacts caused by phase errors, interference, and effects inherent to the magnetic resonance imaging system. It includes ghosting associated with periodic motion or flow, gradient, and eddy current failures, as well as RF contamination—for example, zipper or herringbone patterns. To address these effects, the literature combines several strategies: (i) gradient calibration and flow-sensitive acquisition protocols; (ii) projections into interference-null subspaces; (iii) iterative reconstructions with constraints; and (iv) k-space filtering. In parallel, deep learning is increasingly used to detect and suppress periodic artifacts by learning feature representations that capture their underlying structure.

In the case of RF interference and “spikes” (zipper/herringbone), which appear as bands or specks due to unwanted RF emissions or signal nonlinearities, classical filtering methods may fall short in emerging systems, such as Radiowave Amplification by Stimulated Emission of Radiation (RASER) MRI, where nonlinear behavior dominates. For this scenario, a two-stage deep learning pipeline has been proposed: first, a convolutional network corrects 1D sinograms, and then a U-Net refines the 2D reconstruction. Training uses synthetic data generated from controlled variations in the theoretical RASER model. These simulations generate responses in different modes and apply transforms (Fourier and Radon) to create distorted images, employing domain randomization to improve robustness. The resulting images are practically independent of the degree of nonlinearity. However, the method critically depends on the validity of the physical model and the number of modes considered, whose computational cost increases sharply, thus limiting its extrapolation to higher resolutions [66].

A hybrid approach based on CNNs and Deep Belief Networks (DBNs) has been applied to reduce metallic artifacts in brain MRI. The system extracts features using the Gray Level Co-occurrence Matrix (GLCM), performs segmentation and morphological operations, and classifies images as normal or tumor-bearing. After artifact removal—guided by improvements in SNR and energy metrics—classification accuracy increases from 92.12% to 95.77%. The DBN phase relies on its characteristic energy function to adjust weights and latent representations. This framework demonstrates clinical viability for tumor diagnosis, but its generalizability may be limited by the need for annotated data, specific segmentations, and the absence of an explicit physical model of the artifacts, making transfer to other types of interference difficult [61]. Further validation with experimental RASER data is needed, along with exploration of hybrid architectures that integrate physical models and optimization of computational efficiency to scale to higher resolutions.

Gradient nonlinearities and eddy currents introduce spatial-frequency deviations that result in geometric distortions and ray-like artifacts. Solutions include gradient response calibration, effective field modeling using direct measurements, and gradient matrix-based corrections, along with image interference suppression or k-space techniques [7]. In this context, ACC improves SNR by combining coils with weights derived from the main component that maximizes SNR, while Beamforming-based STreak Artifact Reduction (B-STAR) maximizes the signal-to-interference ratio using a global interference correlation matrix [63]. More recently, Cancellation of streAk artifaCts using the inTerference nUll Space (CACTUS) identifies a low-dimensional interference subspace from the spectral decomposition of the interference correlation matrix and projects the coil data onto the orthogonal complement of this subspace. This approach, which is invariant in k-space and compatible with iterative reconstructions, enhances the cancellation of artifacts caused by gradient nonlinearity without amplifying noise. Practical questions remain, such as its robustness to different levels of subsampling and the optimal choice of the dimension of this subspace [67].

Flow- or pulsatility-induced ghosting, common in Epithelial Inflammatory Disease (EID) and especially relevant in the left hepatic lobe, has been mitigated using acquisition strategies (such as gradients that cancel the first moment) and with classical post-processing methods (outlier exclusion, weighted averaging, or percentile-based approaches). These techniques usually improve lesion–background contrast, although they sometimes attenuate vascular darkness. As an alternative, a feature-driven U-Net has been proposed that is not trained against a gold-standard image, but instead optimizes four normalized scores: pulsation artifacts, vascular darkness, contrast-to-noise ratio (CNR), and data consistency. The goal is to combine the average of these metrics with additional constraints. These terms stabilize variance in the reconstruction, penalize residual pulsation, and preserve image fidelity. Compared with the best conventional strategy, this approach improves image quality, increases CNR, and reduces artifacts without sacrificing vascular darkness [68].

Additionally, cerebrospinal fluid flow artifacts in cervical MRI were reduced using a CycleGAN model trained with T2 TSE (artifact-free reference) and T2 Fast-Field Echo (FFE) images [69].

Table 1 provides a consolidated overview of recent deep learning-based strategies for artifact correction in medical imaging. For each major artifact family, we summarize the typical acquisition- and system-related causes, the predominant methodological directions proposed in the literature, and the main limitations that currently constrain robustness, generalization, and clinical deployment.

Although previous studies have reported effective MRI artifact correction, as in our proposal, most have focused on broader reconstruction problems or non-periodic degradations. Many also rely on dual-domain designs, acquisitions with field maps or reversed polarity, or other strategies that increase methodological and computational complexity. In contrast, our work specifically addresses periodic artifacts by combining two strategies: Radon transform-guided orientation normalization before training, which reduces pattern variability, and wavelet-domain correction using DWT sub-bands with a band-weighted loss that emphasizes the high-frequency components, where these artifacts are most prominent.

## 3. Materials and Methods

### 3.1. Dataset

In this study, we used structural magnetic resonance imaging data from the Dallas Lifespan Brain Study (DLBS) [70]. The dataset comprises 967 high-resolution T1-weighted volumes with isotropic 1 mm voxels.

MRI acquisitions were performed on a Philips Achieva 3T scanner (Philips Healthcare, Best, The Netherlands) equipped with an 8-channel head coil. A 3D Magnetization-Prepared Rapid Acquisition with Gradient Echo (MPRAGE) sequence was used (TR = 8.1 ms, TE = 3.7 ms, rotation angle = 12°, Field of View (FOV) = 204 × 256 mm^2^, and 160 slices), resulting in volumes of 160 × 256 × 256 voxels. The cohort comprised approximately 500 healthy participants aged between 20 and 90 years.

### 3.2. Artifact Generation

This section summarizes the origin of these artifacts, their image manifestations, and, when relevant, their k-space signatures. It also describes the reproducible protocols for generating ringing, herringbone, and zipper artifacts.

MRI is based on nuclear magnetic resonance [71], and due to the complexity of MRI acquisition, several types of artifacts may arise. In this work, we group them according to their origin: (i) acquisition, sampling, and reconstruction effects [72,73]; (ii) patient or physiological motion [74,75,76,77,78]; (iii) $B_{0}$ inhomogeneity and susceptibility effects, which are frequent in the presence of metallic implants [74,79]; and (iv) hardware or RF interference, which can produce zipper and herringbone patterns [79,80].

#### 3.2.1. Ringing

The ringing artifact manifests as edge oscillations (light and dark bands) around high-contrast transitions. It is mainly caused by the truncation of high-frequency components in k-space (finite sampling) or by windowing during reconstruction [81].

Reproducible generation model

To generate ringing artifacts in a reproducible manner, a brain MRI volume $f\left(x,y,z\right)$ is first transformed into k-space by applying a 3D discrete Fourier transform (Equation (1)):

(1) $$F\left(u,v,w\right)=\sum_{z=0}^{N_{z}-1}\sum_{y=0}^{N_{y}-1}\sum_{x=0}^{N_{x}-1}f\left(x,y,z\right)e^{-j2\pi \left(\frac{ux}{N_{x}}+\frac{vy}{N_{y}}+\frac{wz}{N_{z}}\right)},\begin{array}{c} u=0,\ldots ,N_{x}-1\\ v=0,\ldots ,N_{y}-1\\ z=0,\ldots ,N_{z}-1 \end{array}$$

After obtaining the k-space representation in (1), the artifact is introduced slice-wise by modifying the 2D Fourier domain of each slice. Specifically, for each 2D slice $f_{z}\left(x,y\right)$, its 2D spectrum is computed as $F_{z}\left(u,v\right)=F_{2D}\left\{f_{z}\left(x,y\right)\right\}$:

(2) $$\alpha =\left(\frac{1}{2}\sin\left(\pi {\left(1-\frac{r_{i}}{r_{e}}\right)}^{0.55}-\frac{\pi }{2}\right)+\frac{1}{2}\right)U,\;U\sim u\left(0,1\right)$$

Then, α is applied only to a sector of the k-space defined by an angular interval $\left[\theta_{1},\;\theta_{2}\right]$ and a radial band $\left[r_{i},\;r_{e}\right]$, as defined by:

(3) $$\tilde{F_{z}}\left(u,v\right)=\left\{\begin{array}{cc} \alpha F_{z}\left(u,v\right), & if\;\theta_{1}\leq \theta \left(u,v\right)\leq \theta_{2}andr_{i}\leq r\left(u,v\right)\leq r_{e}\\ F_{z}\left(u,v\right), & otherwise \end{array}\right.$$

where $r\left(u,v\right)$ and $\theta \left(u,v\right)$ are computed with respect to the k-space center $\left(u_{0},v_{0}\right)$:

(4) $$r\left(u,v\right)=\sqrt{{\left(u-u_{0}\right)}^{2}+{\left(v-v_{0}\right)}^{2}},\;\;\theta \left(u,v\right)=\mathrm{atan}2\left(v-v_{0},u-u_{0}\right)$$

As a result, two symmetric angular sectors of k-space are modified, producing oscillatory patterns in the spatial domain that characterize the ringing effect. Finally, the corrupted volume is obtained by applying the inverse 3D Fourier transform:

(5) $$f\left(x,y,z\right)=\frac{1}{N_{x}N_{y}N_{z}}\;\sum_{w=0}^{N_{z}-1}\sum_{v=0}^{N_{y}-1}\sum_{u=0}^{N_{x}-1}F\left(u,v,w\right)e^{+j2\pi \left(\frac{\mathrm{ux}}{N_{x}}+\frac{\mathrm{vy}}{N_{y}}+\frac{\mathrm{wz}}{N_{z}}\right)}$$

##### Control Parameters

To control the severity of the artifact, the following parameter ranges were selected empirically (Table 2). Intensity $\alpha \;\in \left[0,\;1\right]$, propagation axis in $\left\{0,\;1,\;2\right\},\;$end angle $\theta_{2}\in \left[0,\;360\right]$, initial angle $\theta_{1}\in \left[\theta_{2}-100,\;\theta_{2}-10\right]$, outer radius $r_{e}\in \left[118,\;123\right]$, and inner radius $r_{i}\in \left[r_{e}-20,\;r_{e}-10\right]$.

#### 3.2.2. Herringbone

This artifact manifests as oblique light-and-dark bands that regularly cross the entire field of view, often near ±0° or ±90°. Its origin lies in the injection of a periodic or coherent signal into the acquisition chain, such as RF interference, hardware failures, or clock desynchronization. This introduces discrete peaks in k-space that are reconstructed as an oblique sinusoidal pattern in the image [82].

##### Generation Model

To generate this artifact, a brain MRI volume $f\left(x,\;y,\;z\right)$ is first converted to k-space by applying the Fourier transform (Equation (1)). The artifact is then introduced slice-wise by modifying the 2D Fourier spectrum of each slice.

For each slice $f_{z}\left(x,y\right)$, we compute $F_{z}\left(u,v\right)=F_{2D}\left\{f_{z}\left(x,y\right)\right\}.$

A local k-space disturbance is created by copying a small kernel centered at the k-space origin $\left(u_{0},\;v_{0}\right)$ to a displaced location $\left(u_{p},\;v_{p}\right)$ and scaling it by a factor β, which is controlled by a smoothing parameter $S\in \left[3,\;20\right]$, thus $\beta =\frac{1}{100s}$.

Let $\Omega_{p}$ be the target kernel region of size $k_{s}\times k_{s}$ centered at $\left(u_{p},\;v_{p}\right)$:

(6) $$\Omega_{p}=\left\{\left(u,v\right):\left|u-u_{p}\right|\leq \frac{k_{s}-1}{2},\left|v-v_{p}\right|\leq \frac{k_{s}-1}{2}\right\}$$

The modified spectrum $\tilde{F_{z}}\left(u,v\right)$ is defined as:

(7) $$\tilde{F_{z}}\left(u,v\right)=\left\{\begin{array}{cc} \beta F_{z}\left(u-\left(u_{p}-u_{0}\right),v-\left(v_{p}-v_{0}\right)\right), & if\;\left(u,v\right)\in \Omega_{p}\\ F_{z}\left(u,v\right), & otherwise \end{array}\right.$$

As a result, a localized perturbation in k-space (a “peak-like” insertion) is produced at a position displaced from the center along either the horizontal or vertical direction. In the spatial domain, this reconstructs as a coherent oblique banding pattern. Finally, the inverse Fourier transform (Equation (5)) is applied to obtain the artifact-corrupted volume.

##### Control Parameters

To control the severity of the artifact, the following parameter ranges were selected empirically (Table 3): smoothing parameter $S\in \left[3,20\right]$, propagation axis in $\left\{0,1,2\right\}$, selection point in $\left\{0,1,2,3\right\}$, kernel size $k_{S}\in \left[3,13\right]$, and displacement distance $d\in \left[-30,30\right]$. The selection point defines the reference location around the k-space center $\left(u_{0},\;v_{0}\right)$, from which the target point $\left(u_{p},\;v_{p}\right)$ is obtained by applying the displacement d along the chosen axis.

#### 3.2.3. Zipper

This artifact appears as one or more lines of noise, often alternating between light and dark pixels, extending across the image in a single direction [83]. In clinical MRI, zipper artifacts may arise from several causes, most commonly related to external RF interference or hardware or software issues outside the radiologist’s direct control.

##### Generation Model

To generate this artifact, for each 2D slice $f_{z}\left(x,y\right)$ of the brain volume, random noise with half intensity is applied to the entire slice. Additionally, if the pixel coordinate falls within the interval defined by a starting position $p_{a}$ and an upper limit $p_{a}+aU$ (where a is the artifact amplitude and $U∼U\left(0,1\right))$, the full noise intensity is injected (Equation (8)).

The interval is evaluated along the selected propagation axis.

Let $n\left(x,y\right)∼U\left(-1,\;1\right)$ be a random noise field and let i be the noise intensity. Let $t\left(x,y\right)$ denote the coordinate of $\left(x,\;y\right)$ along the propagation axis. The corrupted slice $f_{z}^{\prime }\left(x,y\right)$ is defined as:

(8) $$f_{z}^{\prime }\left(x,y\right)=\left\{\begin{array}{cc} f_{z}\left(x,\;y\right)+in\left(x,y\right), & if\;p_{a}\leq t\left(x,y\right)\leq p_{a}+aU\\ f_{z\left(x,\;y\right)}+\frac{i}{2}n\left(x,\;y\right), & otherwise \end{array}\right.,\;U\sim u\left(0,1\right)$$

As a result, a stripe-like noise variation appears in one direction, producing the characteristic zipper artifact pattern.

##### Control Parameters

To control the severity of the artifact, the following parameter ranges were selected empirically (Table 4). The intensity ranged from 15 to 50, the propagation axis from 0 to 2, the number of artifacts from 1 to 16, the variability from 20 to 40, and the amplitude from 10 to 50.

### 3.3. Preprocessing

For volume preprocessing, intensity standardization was applied using z-score normalization, Z=(X−μ)/σ, where X denotes the original voxel intensity, μ is the mean intensity, and σ is the corresponding standard deviation. This transformation centers the intensities at zero and rescales them to unit variance, facilitating inter-image comparison and stabilizing training.

The 3D volumes were divided into 2D slices, and the dataset was partitioned into training (80%) and validation (20%) sets using stratified sampling by artifact type. In addition, the train–validation split was performed at the subject level before any 2D slicing, ensuring that all slices from a given subject or volume were assigned exclusively to one split.

### 3.4. Validation of Dataset Partitioning and Distribution

This section evaluates whether the sets follow comparable distributions, ensuring that the model is exposed to a similar level of complexity in both partitions. First, we assessed whether the proportion of samples for each artifact type remained approximately constant across the training and validation subsets, thereby maintaining balanced representation of artifact classes. Second, we compared the statistical characteristics of the input images between the two subsets to confirm that the data complexity is similar in both partitions.

#### 3.4.1. Feature Extraction Within a Tissue Mask

For each 2D slice, an automatic tissue mask was generated using intensity percentiles to remove background and restrict feature computation to the tissue region. From the voxels inside the mask, we extracted a feature vector composed of the following:

(i) Intensity descriptors, including the mean, standard deviation, skewness, kurtosis, selected percentiles, and a 64-bin histogram computed in z-score space.

(ii) Spatial-frequency descriptors, derived from wavelet- and Fourier-domain energy ratios. Using a first-level 2D discrete wavelet transform with a Daubechies-2 wavelet, we computed the average energies of the sub-bands AA, AD, DA, and DD. We also calculated the ratio between high- and low-frequency energy regions in the Fourier spectrum. The resulting frequency feature vector is defined as:

(9) $$f^{freq}=\left[\frac{E_{AD}+E_{DA}+E_{DD}}{E_{AA}+\epsilon },\;\frac{E_{high}}{E_{low}+\epsilon }\right]$$

where $E_{AA},E_{AD},E_{DA},E_{DD}$ denote the average energies of the level-1 wavelet sub-bands, and $E_{low},E_{high}$ denote the average energies of the low- and high-frequency regions of the Fourier magnitude spectrum. Here, ϵ>0 is a small constant introduced to avoid division by zero.

(iii) Second-order texture descriptors were derived from gray-level co-occurrence matrices (GLCMs). Each image was quantized into 32 gray levels, and the corresponding GLCMs were computed and averaged across four orientations (0°, 45°, 90°, and 135°). The resulting texture feature vector is defined as:

(10) $$f^{glcm}\left(P\right)=\left[\sum_{i,j}{\left(i,\;j\right)}^{2}P_{ij},\;\;\sum_{i,j}\frac{P_{ij}}{1+\left|i-j\right|},\;\sum_{ij}P_{ij}^{2},\;\;\frac{\sum_{i,j\;}\left(i-\mu_{x}\right)\left(j-\mu_{y}\right)P_{ij}}{\sigma_{x}\sigma_{y}+\epsilon },\;\;\sum_{i,j}\left|i-j\right|P_{ij}\right]$$

where $P_{ij}\;$ denotes the normalized GLCM averaged across the four orientations, and $\mu_{x},\mu_{y},\sigma_{x},\sigma_{y}$ denote the marginal means and standard deviations of P, computed from the row and column marginals.

#### 3.4.2. Joint Normalization and Decorrelation

After feature extraction, feature vectors from the training and validation partitions were jointly normalized. First, a pooled z-score normalization was applied to each feature dimension by subtracting the global mean and dividing by the pooled standard deviation. Next, a whitening transformation based on the pooled covariance matrix was applied to reduce linear correlations between dimensions.

This procedure produces a multivariate representation with approximately zero-mean, unit variance, and reduced cross-feature correlations.

#### 3.4.3. Multivariate Comparison Tests

The training and validation distributions were compared using three multivariate two-sample tests applied to the normalized feature vectors:

(11) $$\varepsilon \left(X,Y\right)=\frac{2}{nm}\sum_{i=1}^{n}\sum_{j=1}^{m}{\left\|x_{i}-y_{i}\right\|}_{2}-\frac{1}{n^{2}}\sum_{i=1}^{n}\sum_{i’=1}^{n}{\left\|x_{i}-x_{i’}\right\|}_{2}-\frac{1}{m^{2}}\sum_{j=1}^{m}\sum_{j’=1}^{m}{\left\|y_{j}-y_{j’}\right\|}_{2}$$

where $X={\left\{x_{i}\right\}}_{i=1}^{n}$ and $Y={\left\{y_{j}\right\}}_{j=1}^{m}$ are the feature samples from the training and validation sets, respectively.

#### 3.4.4. Maximum Mean Discrepancy (MMD) with a Gaussian Kernel

(12) $${MMD}^{2}\left(X,Y\right)=\frac{1}{n\left(n-1\right)}\sum_{i\neq i’}k_{\sigma }\left(x_{i},x_{i’}\right)+\frac{1}{m\left(m-1\right)}\sum_{j\neq j’}k_{\sigma }\left(y_{j},y_{j’}\right)-\frac{2}{nm}\sum_{i=1}^{n}\sum_{j=1}^{m}k_{\sigma }\left(x_{i},y_{j}\right)$$

with the Gaussian kernel $k_{\sigma }\left(x,y\right)=\exp\left(-\frac{{∥x-y∥}_{2}^{2}}{2\sigma^{2}}\right).$

The bandwidth σ  was selected using the median heuristic, i.e., as the median of pairwise Euclidean distances computed over the pooled set X∪Y.

#### 3.4.5. Sliced-Wasserstein Distance (Order 1)

(13) $${SWD}_{1}\approx \frac{1}{L}\sum_{i=1}^{L}\left[\frac{1}{k}\sum_{k=1}^{K}\left|{\tilde{x}}_{k}^{\left(l\right)}-{\tilde{y}}_{k}^{\left(l\right)}\right|\right],\;\;\;K=\min\left(n,m\right)$$

where $\theta_{l}\in S^{d-1}$ are L random unit directions, and $\left\{{\tilde{x}}_{k}^{\left(l\right)}\right\}$ and $\left\{{\tilde{y}}_{k}^{\left(l\right)}\right\}$ are the sorted one-dimensional projections of X and Y onto $\theta_{l}$.

In all cases, significance was assessed using a stratified permutation test comparing the training and validation sets, yielding *p*-values associated with the null hypothesis that both partitions are drawn from the same multivariate feature distribution. High *p*-values indicate that no statistically significant differences were detected between partitions with respect to the intensity, texture, and spatial-frequency properties considered.

### 3.5. Proposed Architecture

The proposed network, WaveletBasedAttention-Net, takes the four sub-bands of a 2D Discrete Wavelet Transform (DWT) {AA, AD, DA, DD} as input. It is trained to explicitly correct each sub-band.

We employ a 2D U-Net with four encoding and decoding levels, symmetric attention-based skip connections, and a bottleneck containing 1024 filters. Each convolutional block is implemented as a DoubleConv module consisting of 3×3 convolutions, LeakyReLU activation (slope = 0.2), and batch normalization. In the encoder, each block is followed by 2 × 2 max pooling, whereas the decoder uses bilinear upsampling with concatenation of the corresponding skip features. A final convolution reconstructs the four wavelet bands.

The WaveletLoss combines L1 reconstruction errors computed independently for each band using configurable weights $\left(w_{\mathrm{AA}},w_{\mathrm{AD}},w_{\mathrm{DA}},w_{\mathrm{DD}}\right)$.

**Figure 1:**
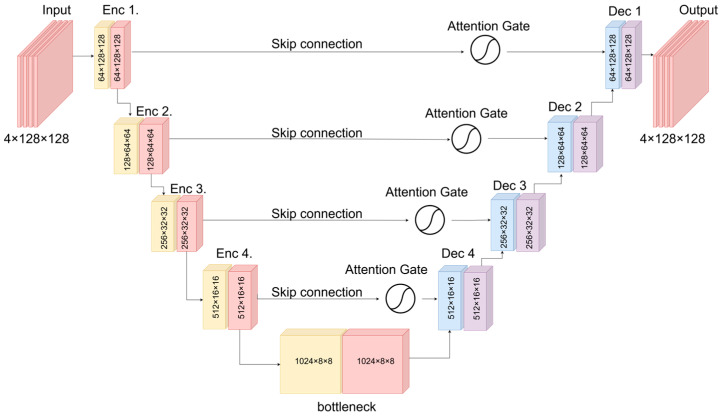
WaveletBasedAttention-Net architecture (DWT→4-band U-Net→IDWT) with hop connections and 1024 filter bottleneck.

Figure 1 summarizes the network topology and tensor dimensions for a typical 4×128×128 tile. Due to the DWT stage followed by four pooling operations, the implementation requires a minimum spatial size of ≥32×32.

**Figure 2:**
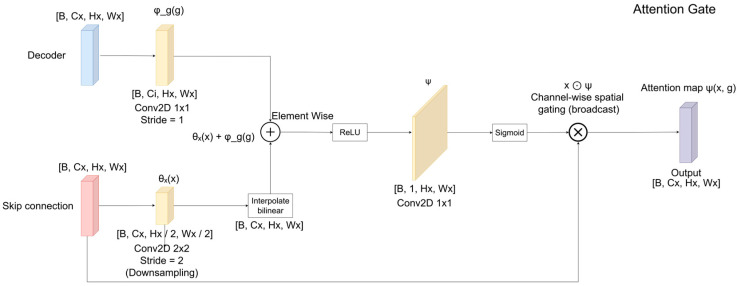
Attention gate (AG) mechanism integrated into the skip connections of the proposed Attention U-Net architecture.

Figure 2 illustrates the attention gate (AG) mechanism integrated into the skip connections of the proposed Attention U-Net architecture [84]. The attention gate selectively emphasizes relevant spatial regions in the encoder feature maps while suppressing irrelevant or noisy activations before feature fusion in the decoder.

Let $x\in \;R^{B\times C_{x}\times H_{x}\times W_{x}}$ denote the encoder feature map (skip connection) and let $g\in R^{B\times C_{g}\times H_{g}\times W_{g}}$ denote the gating signal from the decoder at a coarser resolution.

First, the encoder features are linearly projected and spatially downsampled using a strided convolution (Equation (14)):

(14) $$\theta_{x}={conv}_{2\times 2,s=2}\left(x\right),\theta_{x}\in R^{B\times C_{i}\times H_{\theta }\times W_{\theta }}$$

where $C_{i}$ is an intermediate channel dimensionality.

Simultaneously, the decoder gating signal is projected into the same intermediate feature space using a 1×1 convolution (Equation (15)):

(15) $$\phi_{g}=\;{conv}_{1\times 1}\left(g\right),\;\;\phi \in R^{B\times C_{i}\times H_{g}\times W_{g}}$$

If necessary, $\phi_{g}$ is spatially interpolated to match the spatial resolution of $\theta_{x}$. The resulting feature maps are then combined through element-wise addition and passed through a Rectified Linear Unit (ReLU) (Equation (16)):

(16) $$f=ReLU(\theta_{x}+\phi_{g})$$

To generate the attention coefficients, a 1 × 1 convolution followed by a sigmoid activation is applied (Equation (17)):

(17) $$\psi =\sigma \left({conv}_{1\times 1}\left(f\right)\right),\;\psi \in R^{B\times 1\times H_{\theta }\times W_{\theta }}$$

The resulting attention map ψ encodes the relevance of spatial locations conditioned on the decoder context. It is then upsampled to match the spatial resolution of the encoder feature maps (Equation (18)):

(18) $$\psi_{↑}=upsample\left(\psi \right),\psi_{↑}\in R^{B\times 1\times H_{x}\times W_{x}}$$

Finally, the attention gate modulates the encoder feature map via element-wise multiplication (Equation (19)):

(19) $$\hat{x}=x⊙\psi$$

where $\hat{x}$ denotes the refined skip features passed to the decoder. The formulation of the attention module is summarized in Equations (20) and (21):

(20) $$\alpha \left(x,g\right)=\sigma \left(\psi^{T}RELU\left(\theta_{x}\left(x\right)+\;\phi \left(g\right)\right)\right)$$

(21) $$\hat{x}=x⊙\alpha \left(x,g\right)$$

### 3.6. Training

The complete pipeline begins with Radon-guided orientation normalization. For each artifacted slice, the dominant angle is estimated within the range [0, 180°), and the slice and its ground truth counterpart are rotated to align the artifact pattern. The predicted output is then rotated back to the original reference frame. After rotation, a 2D DWT is applied to obtain the four sub-bands {AA, AD, DA, DD}, which form the input tensor [B, 4, H/2, W/2] for the U-Net. The network predicts corrected bands, and the denoised image is reconstructed using IDWT.

**Figure 3:**
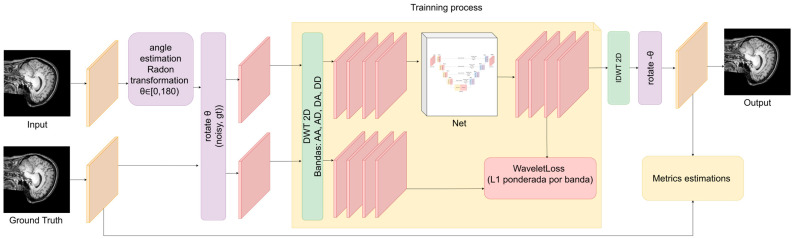
Training and evaluation pipeline: Radon-guided rotation, DWT decomposition, wavelet-based network processing, IDWT reconstruction, and inverse rotation. Loss is computed per band, and metrics are evaluated on the reconstructed image.

During training, optimization is performed exclusively in the wavelet band domain with WaveletLoss (a band-weighted L1 loss), while SSIM is monitored as a structural metric without contributing to the gradient. During validation, in addition to total loss, band-wise metrics (e.g., MAE for DA) and global metrics computed on the spatial reconstruction are reported. The data loaders are configured with explicit batch size and worker settings (Figure 3).

#### 3.6.1. Wavelet Component Selection for Training

This section focuses on selecting the Daubechies (db) wavelet family that yields the best average performance after 5 runs using a U-Net architecture. Each wavelet family decomposes the image using different low-pass and high-pass filters, separating the signal into low- and high-frequency components [85].

The wavelet function can be expressed in terms of the scaling function ∅(t) (Equation (22)):

(22) $$\phi_{L}\left[t\right]=\sqrt{2}\sum_{k=0}^{2L-1}1\left[k\right]\phi_{L}(2t-k)$$

where $I_{k}$ are the scale coefficients and L is the Daubechies order.

The wavelet filter coefficients h[k], which are related to $l_{k}$, are defined as Equation (23):

(23) $$h\left[k\right]={\left(-1\right)}^{k}I\left[2L-1-k\right]$$

Accordingly, the wavelet function can be written as Equation (24):

(24) $$\psi_{L}\left(t\right)=\sqrt{2}\sum_{k=0}^{L-1}h\left[k\right]\phi_{L}(2t-k)$$

For each component $\left\{db_{1},\ldots ,db_{6}\right\}$, both the number of filters and the coefficients vary. The number of filter coefficients is equal to twice the order of the wavelet. Thus, $db_{1}$ contains two filter coefficients, whereas $db_{6}$ contains twelve.

#### 3.6.2. Loss Function (WaveletLoss)

Training optimizes a loss function defined in the wavelet domain that weights the error of each band to direct the correction towards the components where artifacts concentrate most of their energy (Equation (25)).

Given the prediction ${\hat{B}}_{b}^{\left(n\right)}$ and the reference $B_{b}^{\left(n\right)}$ for band $b\in \left\{\mathrm{AA},\mathrm{AD},\mathrm{DA},\mathrm{DD}\right\}$ of slice n, the loss is defined as the mean L1 error computed independently for each band. Each band contribution is weighted by the corresponding coefficient $w_{b}$:

(25) $$L_{wavelet}=\sum_{b\in \left\{AA,AD,DA,DD\right\}}W_{b}\frac{1}{NH_{b}W_{b}}\sum_{n=1}^{N}{\left\|{\hat{B}}_{b}^{\left(n\right)}-B_{b}^{\left(n\right)}\right\|}_{1}$$

In the implementation, each term is computed over the batch and spatial dimensions, then linearly combined using the band weights. The loss operates exclusively on the four bands produced by the 2D DWT, while the image reconstructed via the IDWT is used only to compute evaluation metrics (e.g., SSIM) that do not backpropagate gradients.

To favor suppression of periodic patterns while preserving low-frequency content, we use by default uneven weights emphasizing the high-frequency bands, $\{w_{\mathrm{AA}},w_{\mathrm{AD}},w_{\mathrm{DA}},w_{\mathrm{DD}}\}\;=\;\left\{0.1,0.3,0.4,0.3\right\}$.

Table 5 summarizes the hyperparameters selected for training.

### 3.7. Comparison of Results

To compare the results, several models were trained, as described below. All baseline models were implemented using a U-Net architecture with the four db2 wavelet sub-bands. The differences between the models lie in the encoder and decoder layers and the types of skip connections used.

#### 3.7.1. U-Net

Among the models considered for comparison is the U-Net, a network originally designed for medical image segmentation [86]. Its use, however, was extended to several other tasks, including image reconstruction [87,88,89] and artifact correction [90,91,92], among others [93,94].

This architecture follows an encoder–decoder structure. The encoder extracts hierarchical image features, while the decoder reconstructs the output according to the target objective, which in this case is artifact correction.

Through its skip connections, U-Net preserves fine spatial details while maintaining anatomical coherence. Moreover, the architecture typically achieves strong performance even when trained with relatively limited amounts of data.

Nevertheless, although the encoder reduces spatial resolution to capture more global contextual information, the model still relies primarily on local receptive fields. Furthermore, some 3D implementations substantially increase the computational cost.

#### 3.7.2. GAN

Another network considered for comparison is the generative adversarial network (GAN) [95], which consists of two components: a generator and a discriminator. The generator produces candidate images, while the discriminator learns to distinguish whether the generated image is consistent with the target distribution. This architecture can model complex data distributions and was widely applied to tasks such as image reconstruction and synthetic data generation. Moreover, GAN-based approaches often produce perceptually more realistic results, avoiding excessively smoothed images.

However, because the generator depends on the discriminator, training can become unstable and difficult to converge. In addition, GANs may introduce structures that are not anatomically faithful, which could negatively affect clinical interpretation.

#### 3.7.3. Spatial and Channel Attention Mechanisms

In addition to the attention-based approach proposed in Section 3.5, we compare two alternative attention mechanisms: spatial attention and channel attention [96]. The spatial attention module focuses on relevant spatial regions and introduces global contextual information into the learned feature maps through a spatial attention map.

Channel attention, in contrast, emphasizes the importance of each feature channel, encouraging the reconstructed features to remain consistent with the input representations. As a result, the encoder outputs are guided to preserve meaningful feature relationships while suppressing less relevant activations. Together, these mechanisms improve the modeling of global dependencies through guided attention. Reported results indicate that such architectures can achieve statistically more robust and accurate predictions.

However, these approaches introduce additional architectural and computational complexity because extra attention operations must be computed during training and inference. Furthermore, they typically require larger training datasets and careful hyperparameter tuning to achieve good generalization.

#### 3.7.4. Attention–Attention

The proposed model relies on a self-attention mechanism in which each image patch attends to all other patches, enabling the modeling of long-range dependencies during reconstruction [97]. Through multi-head self-attention, the encoder captures contextual relationships between degraded and informative regions, allowing the decoder to reconstruct each patch using both local information and global image context.

This mechanism improves robustness and reconstruction quality compared with purely local convolutional approaches. However, the attention mechanism also increases computational complexity and may, in some cases, attend to irrelevant regions, leading to reconstruction errors when foreground and background features are not clearly distinguishable.

#### 3.7.5. Vision Transformer

Another model considered for comparison is the Vision Transformer (ViT) [98], which processes the input image by dividing it into fixed-size patches that are linearly embedded and augmented with positional information. Through a self-attention mechanism, ViT models interactions among all patches, enabling the network to capture long-range global dependencies across the entire image.

This architecture is particularly effective for tasks requiring global contextual reasoning, such as image reconstruction and artifact correction, as it enables more coherent modeling of spatial relationships compared with purely convolutional approaches.

However, ViT-based models typically require larger training datasets to generalize effectively and incur higher computational and memory costs due to the quadratic complexity of the attention mechanism. Additionally, the lack of strong inductive biases, such as locality and translation invariance, may reduce performance in scenarios with limited data.

## 4. Results

This section presents the results obtained in a controlled synthetic evaluation setting, including artifact generation, validation of the synthetic dataset, training outcomes, and visual examples of artifact correction produced by the proposed method.

### 4.1. Artifact Generation

Following the procedure described in Section 2.3, we generated three types of artifacts: ringing, herringbone, and zipper. For each artifact type, we present the original artifact-free image, the corresponding k-space (when applicable), and the visual appearance of the generated artifact in the reconstructed image.

#### 4.1.1. Ringing

**Figure 4:**
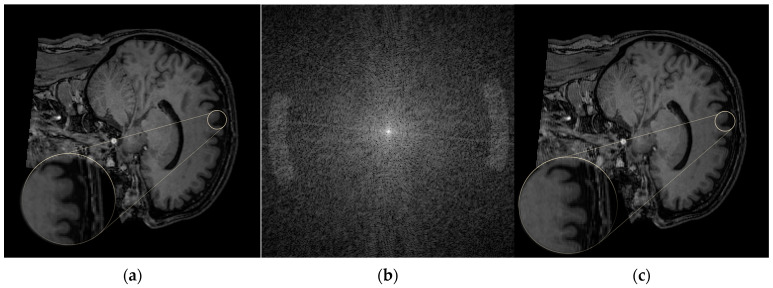
Ringing artifact: image appearance and k-space signature. (**a**) Artifact-free brain MRI. (**b**) k-space signature showing the magnitude spectrum of the artifact component, computed as log (1 + $\left|\;F\;\right|)$. (**c**) Image with the ringing artifact; ellipses show edge-aligned oscillations adjacent to a high-contrast boundary.

Figure 4 illustrates the ringing artifact. Figure 4a shows the original artifact-free image. In Figure 4c, the ringing artifact appears as fine oscillatory bands parallel to image edges and aligned with the sampling direction (readout or phase-encoding direction). The artifact becomes particularly visible at high-contrast interfaces, such as the boundary between cerebrospinal fluid (CSF) and gray matter.

Figure 4b shows the corresponding k-space representation. The artifact originates from variations in the high-frequency components of k-space. In the FFT domain, the spectrum shows concentrated energy near the center, with residual oscillatory patterns corresponding to the undulations observed in the reconstructed image.

#### 4.1.2. Herringbone

**Figure 5:**
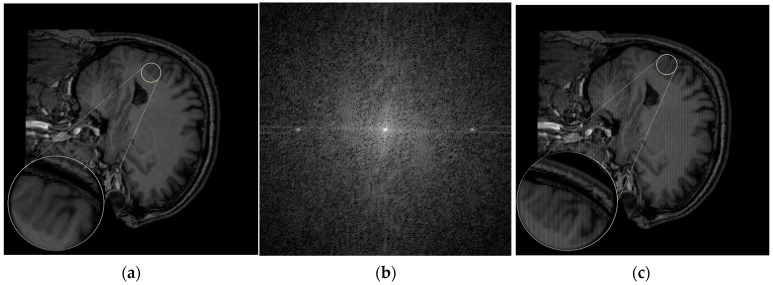
Herringbone artifact: image appearance and k-space signature. (**a**) Artifact-free brain MRI. (**b**) k-space signature showing the log-magnitude spectrum of the artifact component computed as log (1 +$\;\left|\;F\;\right|).$ (**c**) Image with herringbone artifact; the ellipse shows the banding pattern in gray matter.

Figure 5 shows the herringbone artifact. Figure 5a presents the artifact-free image, while Figure 5c displays the image with the herringbone artifact, which appears as thin vertical bands arranged in parallel. These bands become more visible at high-contrast interfaces, such as between CSF and gray matter.

Figure 5b shows the corresponding k-space representation. The artifact component reflects variations in the high-frequency region, and its Fast Fourier transform (FFT) exhibits concentrated energy at the center, with residual dot-like patterns corresponding to the bands observed in the spatial image.

#### 4.1.3. Zipper

**Figure 6:**
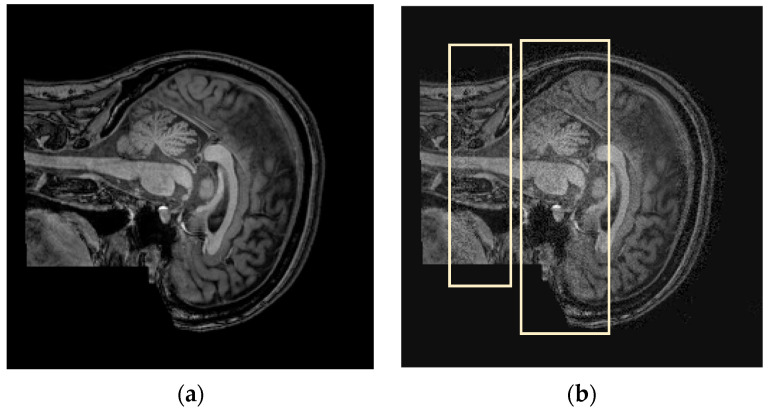
Zipper artifact: image appearance. (**a**) Artifact-free brain MRI. (**b**) Image with the zipper artifact; rectangles show the vertical noise patterns.

Figure 6 shows the zipper artifact. Figure 6a presents the artifact-free image, while Figure 6b displays the zipper artifact, which appears as vertical noise that degrades image quality.

### 4.2. Validation of Dataset Partitioning and Distribution

This section reports the results of validating the dataset partitioning and distribution. The analysis follows the methodology described in Section 3.4.

#### 4.2.1. Distribution of Artifact Types Across Dataset Partitioning

**Figure 7:**
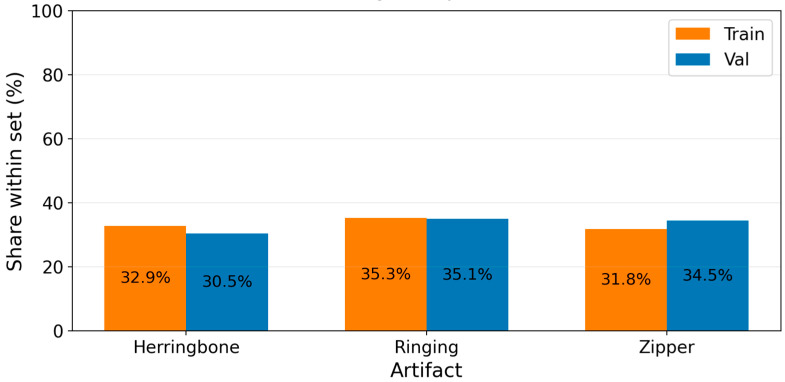
Percentage distribution of artifact types (ringing, herringbone, and zipper) across the training and validation partitions.

We first evaluated the proportion of samples for each artifact type (ringing, herringbone, and zipper) in the training and validation sets. As shown in Figure 7, the proportions remain nearly constant across the two partitions.

Specifically, the herringbone class represents 32.9% of the samples in the training set and 30.5% in the validation set. The ringing artifact accounts for 35.3% and 35.1% of the samples in the training and validation sets, respectively, while the zipper artifact represents 31.8% and 34.5%, respectively. These minor differences indicate that the class distribution remains balanced between the two subsets, reducing the risk of sampling bias during model training and evaluation.

#### 4.2.2. Validation of Dataset Partition Distribution

**Figure 8:**
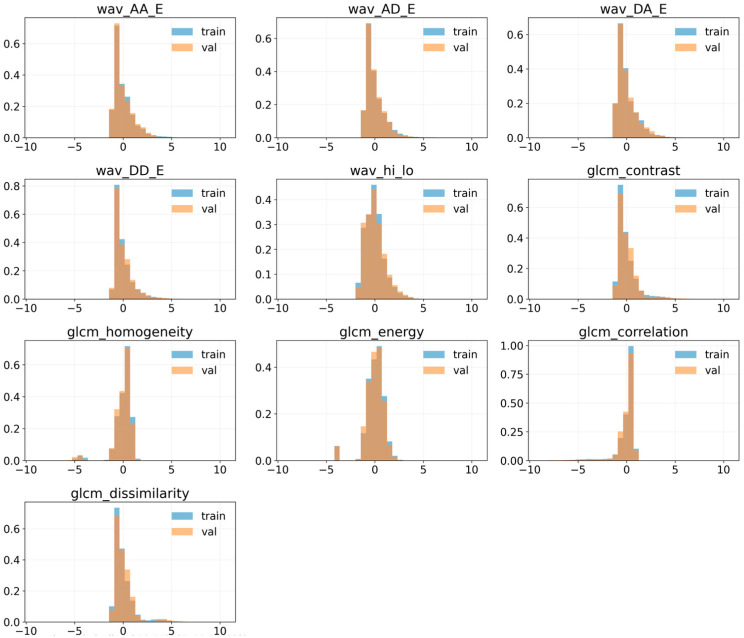
Distribution of wavelet and GLCM descriptors in the training and validation partitions.

Figure 8 shows the Z-score distributions of the wavelet-energy and GLCM texture descriptors for the training and validation partitions. In all cases, the histograms of both subsets largely overlap and exhibit very similar shapes, tails, and amplitudes.

The wavelet sub-band energies and the high-to-low-frequency ratio exhibit strongly right-skewed distributions. In contrast, the GLCM descriptors show consistent patterns across the two partitions: contrast and dissimilarity are concentrated at low values, whereas homogeneity, energy, and correlation remain close to 1.

This visual similarity indicates that both partitions capture comparable frequency and texture characteristics, with no noticeable bias in statistical complexity between the training and validation sets.

**Figure 9:**
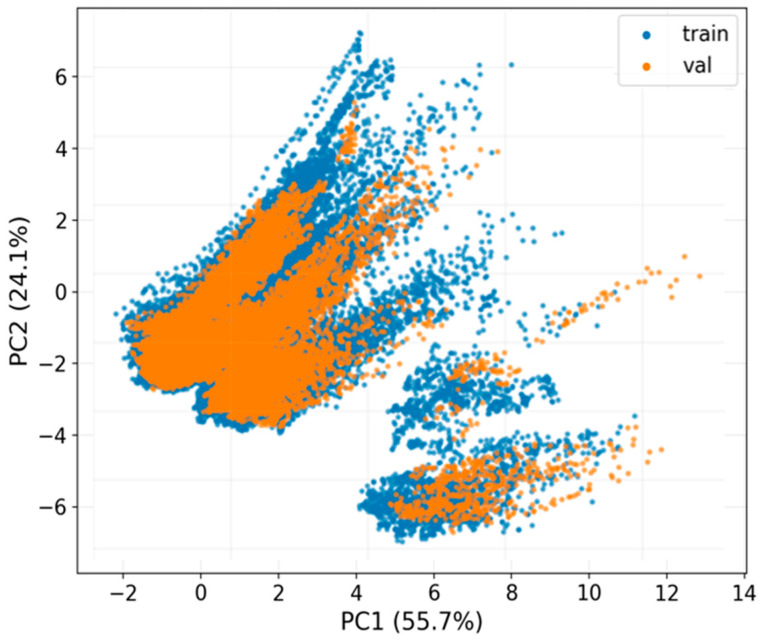
PCA projection of wavelet + GLCM features: training vs. validation distribution comparison.

Furthermore, Figure 9 shows the projection of the feature vectors (wavelet + GLCM) onto the first two principal components (PC1 and PC2), which together explain approximately 80% of the total variance. The samples from the training and validation sets are highly intermingled in the latent space, forming point clouds with very similar shapes and extents. No systematic separation is observed between the two partitions; instead, both occupy the same high-density regions and exhibit similar clustering patterns. This strong overlap indicates that, in terms of frequency and texture features considered, the training and validation distributions are comparable and do not introduce any evident bias into the feature space.

Table 6 summarizes the results of the multivariate analyses performed on the feature subspace defined by wavelet energies and GLCM descriptors using the training and validation partitions. Three statistical tests were applied: Energy Distance, Maximum Mean Discrepancy with a radial basis function kernel (MMD-RBF), and Sliced-Wasserstein distance. The resulting statistics are small (0.01038, 0.00032, and 0.07342, respectively), with *p*-values below 0.05. However, the feature histograms show substantial overlap, and the PCA projection does not reveal a clear separation between the partitions. Therefore, although differences are detected, they do not suggest a severe imbalance between the sets. We believe these differences can be explained by the volume-level partitioning strategy used to prevent data leakage, which naturally introduces minor differences between subsets while preserving methodological validity.

### 4.3. Wavelet Family Selection

To determine the wavelet family used in the subsequent experiments, we conducted an exploratory study on a 5% subset of the full dataset. In this scenario, six Daubechies families (db1–db6) were compared while keeping the network architecture, training hyperparameters, and all other model settings constant.

For each wavelet family, the model was trained for 20 epochs, and the experiment was repeated five times with different random seeds to account for variability arising from initialization and mini-batch sampling. In each run, we recorded the minimum validation loss and the corresponding evaluation metrics (SSIM and MAE in the image domain) at the epoch with the lowest validation loss.

The results were then aggregated as mean ± standard deviation for each wavelet family, as summarized in Table 7.

Among the evaluated wavelet families, db2 achieved the highest average validation SSIM. As training cost scales with dataset size and available computational resources, repeating full-dataset training for each wavelet family would be computationally expensive. Therefore, this wavelet-selection study was conducted on a reduced subset of the complete dataset and limited to five independent runs per wavelet, providing a practical compromise between robustness and computational cost.

Given this limited number of runs, the experiment does not provide sufficient statistical power to claim differences between wavelet families. Accordingly, the observed differences should be interpreted as indicative rather than conclusive statistical evidence. Based on these results, the db2 wavelet family was selected for all subsequent experiments.

### 4.4. Training Results

This section reports the loss, SSIM, PSNR, and MAE results obtained for each dataset split. For the training set, the reported values correspond to the model’s best epoch.

**Figure 10:**
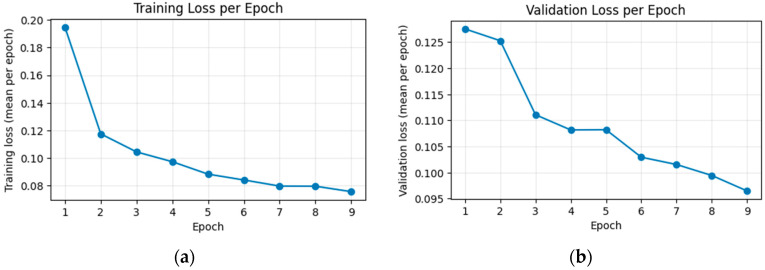
Evolution of the compound loss $w_{AA},w_{AD},w_{DA},w_{DD}$ across epochs for the training and validation partitions. (**a**) Training loss; (**b**) validation loss.

Figure 10 illustrates the evolution of the compound loss $w_{AA},w_{AD},w_{DA},w_{DD}$ as a function of epoch for both the training and validation partitions. In the training set, the loss decreases sharply, dropping from approximately 0.198 in the first epoch to around 0.078 in the final epoch. This trend indicates that the model progressively adjusts its parameters to the characteristics of the training data.

In the validation set, the loss starts at approximately 0.130 and converges to around 0.097 by the ninth epoch. Although small oscillations are observed between epochs, the validation loss remains within a bounded range and does not exhibit sustained increases. This suggests that the model does not show pronounced overfitting and that its generalization performance remains stable throughout training.

**Figure 11:**
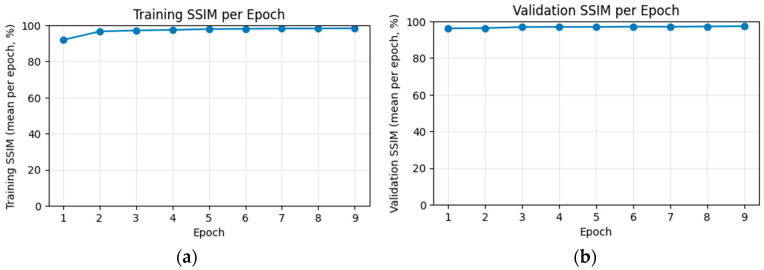
Evolution of the SSIM metric across epochs for the training and validation partitions. (**a**) Training SSIM; (**b**) validation SSIM.

Figure 11 shows the evolution of the structural similarity index (SSIM) for the training and validation partitions over nine epochs. In the training set, the mean SSIM increases progressively from 91.8% in the first epoch to 98.3% in the ninth epoch, reflecting a steady improvement in reconstruction quality.

In the validation set, SSIM remains consistently high throughout training, with moderate fluctuations between approximately 96% and 97%. It increases from 96.2% in the first epoch and stabilizes around 97.4% by the last epoch.

The close alignment between the training and validation curves, without systematic degradation on the validation set, indicates that the model generalizes well and does not exhibit significant overfitting with respect to SSIM.

**Figure 12:**
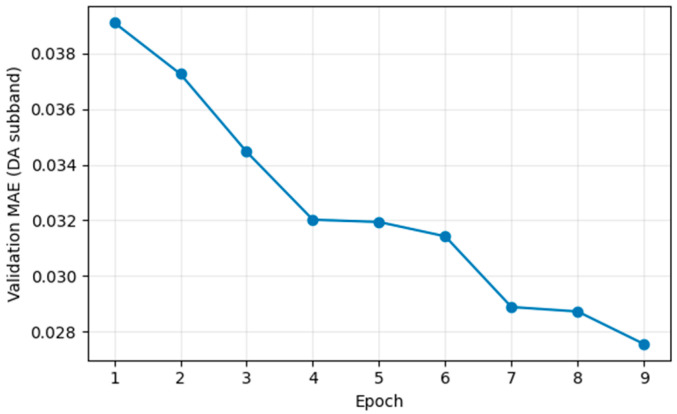
Evolution of the validation MAE across epochs, computed on the high-frequency DA wavelet sub-band.

Figure 12 shows the evolution of the validation MAE calculated on the DA wavelet sub-band. The error decreases from approximately 0.039 in the first epoch to around 0.027 by the ninth, indicating a progressive and relatively stable improvement during training.

This metric is evaluated specifically on the high-frequency component because the artifacts of interest (ringing, herringbone, and zipper) are most pronounced in these sub-bands, along with the fine edge and texture details that are diagnostically relevant. In contrast, the low-frequency components (such as AA) are dominated by the overall structure and coarse contrast, which are typically easier to reconstruct and less sensitive to small distortions.

By focusing the MAE on the DA sub-band, the metric provides a more targeted assessment of correction quality in the regions where artifacts primarily affect high-frequency information. This allows us to assess whether the model suppresses these distortions while preserving subtle anatomical details.

**Figure 13:**
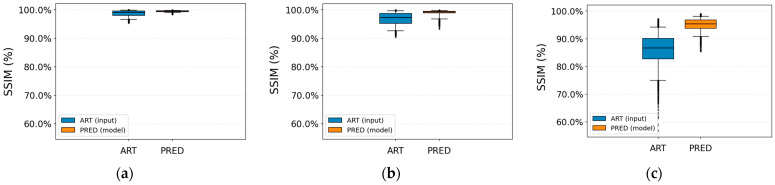
SSIM results by artifact type before and after correction. (**a**) Ringing artifact; (**b**) herringbone artifact; (**c**) zipper artifact.

**Figure 14:**
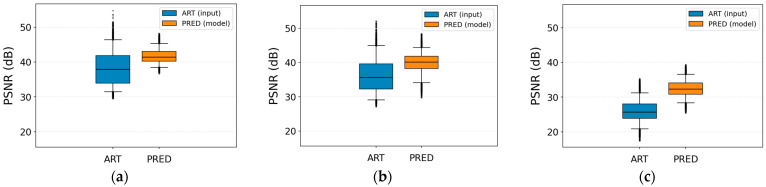
PSNR before and after correction for each artifact type. (**a**) Ringing artifact; (**b**) herringbone artifact; (**c**) zipper artifact.

Figure 13 and Figure 14 show consistent improvements in SSIM and PSNR after correction for all three artifact types. For the herringbone artifact, the average SSIM increases from 0.968 ± 0.0228 to 0.99 ± 0.0096 (approximately +2.2 percentage points), while the PSNR improves from 36.166 ± 4.893 dB to 39.892 ± 3.046 dB.

For ringing, the input SSIM is already very high (0.987 ± 0.011) but still increases to 0.995 ± 0.003, accompanied by an improvement in PSNR from 38.17 ± 4.824 dB to 41.658 ± 2.062 dB.

The zipper artifact shows the largest improvement, with SSIM increasing from 0.860 ± 0.059 to 0.951 ± 0.023, and PSNR rising from 25.958 ± 3.186 dB to 32.424 ± 2.427 dB. These results indicate that the proposed model is particularly effective and consistent in correcting this type of artifact.

**Figure 15:**
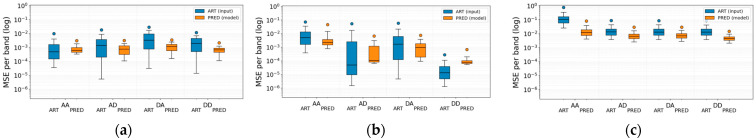
MSE by wavelet band before and after correction for each artifact type. (**a**) Ringing artifact; (**b**) herringbone artifact; (**c**) zipper artifact.

Figure 15 shows the MSE values per wavelet band (AA approximation and AD, DA, and DD details) for each artifact type, comparing images with artifacts (ART) and model-corrected images (PRED). The logarithmic MSE scale shows a consistent shift towards lower values after correction, particularly in the high-frequency bands.

For ringing, the AD, DA, and DD detail components show a clear reduction in MSE and reduced dispersion, indicating that the model consistently attenuates the edge oscillations associated with this artifact. A similar pattern is observed for herringbone: the error decreases substantially in the detail bands, and the distributions become narrower, while the approximation component remains relatively stable. This suggests that the correction mainly targets high-frequency periodic structures.

Finally, for the zipper artifact, the reduction in MSE is particularly pronounced across all bands, with lower medians and more compact interquartile ranges. This indicates that the model effectively removes much of the error energy associated with interference lines, preserving low-frequency structural information.

Table 8 summarizes the quality metrics before and after artifact correction. A reduction in the MSE is observed across all weighted wavelet sub-bands. Specifically, in the $w_{AA}$ component, the MSE decreases from 0.048 ± 0.869 to 0.007 ± 0.009, in $w_{AD}$ from 0.008 ± 0.011 to 0.003 ± 0.004, in $w_{DA}$ from 0.009 ± 0.011 to 0.004 ± 0.004, and in $w_{DD}$ from 0.007 ± 0.010 to 0.002 ± 0.003. Notably, the unusually large pre-correction dispersion in MSE_wAA reflects a small subset of severely corrupted slices in which the periodic artifact contributes substantial energy to the approximation (AA) band, producing a strongly right-skewed distribution rather than a numerical inconsistency.

The weighted-average MSE decreases from 0.018 ± 0.029 to 0.004 ± 0.005. These improvements are also reflected in the perceptual quality metrics: the average PSNR increases from 33.42 ± 6.939 dB to 43.337 ± 5.364 dB, while the SSIM rises from 0.938 ± 0.067 to 0.985 ± 0.022. These results indicate that the corrected images exhibit lower distortion and greater structural similarity to the artifact-free references.

### 4.5. Structural Preservation and Edge Integrity

To assess whether artifact suppression preserves anatomical structures, we complemented standard image-quality metrics with gradient- and edge-based measures computed slice-wise. Images were first evaluated in the same z-score space used for model inference, ensuring consistent intensity scaling between input, prediction, and ground truth. In addition to PSNR and SSIM in the image-intensity domain, we computed metrics on Sobel-gradient magnitude maps to assess edge consistency. We also measured gradient errors (L1 and L2) between predicted and ground truth gradients to quantify structural deviations. Edge Dice (F1) was obtained from binary edge maps derived from Sobel magnitude using a robust threshold based on the 90th percentile of the ground truth gradient distribution. These complementary metrics serve as proxies for anatomical structural integrity and boundary preservation, helping distinguish true structural recovery from simple image smoothing. In addition, we evaluated a Radon-based periodicity proxy on residual error maps to determine whether oriented artifact patterns remain after correction.

The proposed method improves image fidelity while maintaining structural consistency. Improvements are observed in the gradient-based domain: Edge-PSNR increases from 38.41 to 41.04, and Edge-SSIM from 0.952 to 0.980, indicating stronger agreement between edge structures and the ground truth images. Gradient mismatch errors decrease (Grad-L1: 0.0744 to 0.0540, Grad-L2: 0.0622 to 0.0436), and edge overlap improves (Dice/F1: 0.920 to 0.938), suggesting that boundary structures are preserved rather than smoothed away. Finally, the Radon peak ratio measured on residual errors decreases from 6.72 to 2.89, indicating a reduction in oriented periodic artifact patterns (Table 9). The relatively large pre-correction variability of this metric reflects a strongly right-skewed distribution driven by a small number of severe slices, in which structured periodic residuals dominate the error map.

### 4.6. Visual Results of the Correction

**Figure 16:**
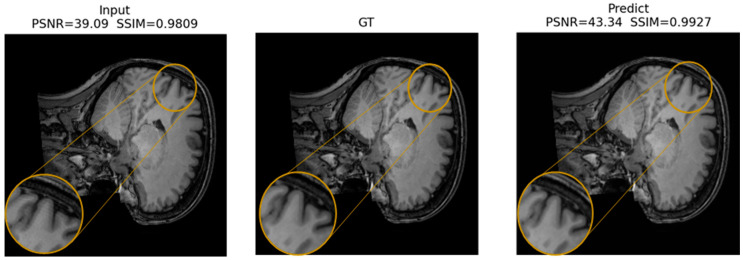

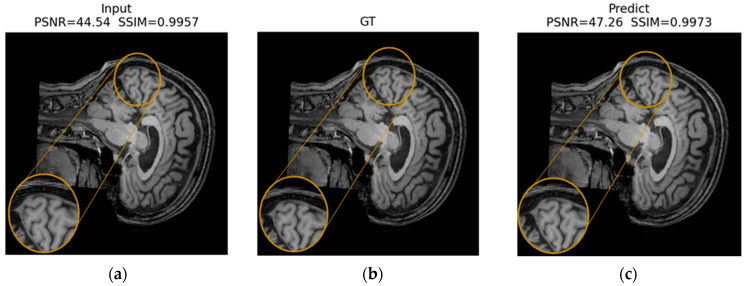
Visual comparison of ringing artifact correction. (**a**) Artifacted input image (**b**), ground truth (GT), and (**c**) model prediction. Highlighted regions show areas affected by the ringing artifact, where the proposed model achieves improved restoration quality, reflected in higher PSNR and SSIM values.

**Figure 17:**
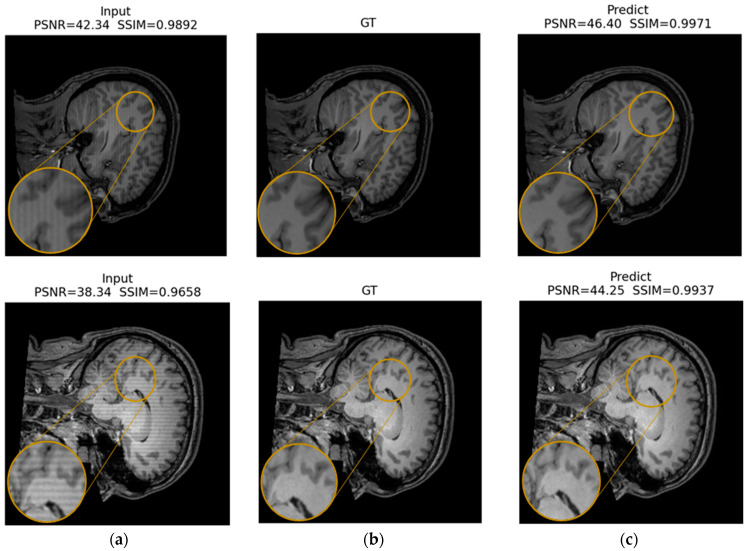
Visual comparison of herringbone artifact correction. (**a**) Artifacted input image; (**b**) ground truth (GT); and (**c**) model prediction. Highlighted regions indicate areas affected by the herringbone artifact, where the proposed model achieves noticeable improvements in image quality, reflected in higher PSNR and SSIM values.

**Figure 18:**
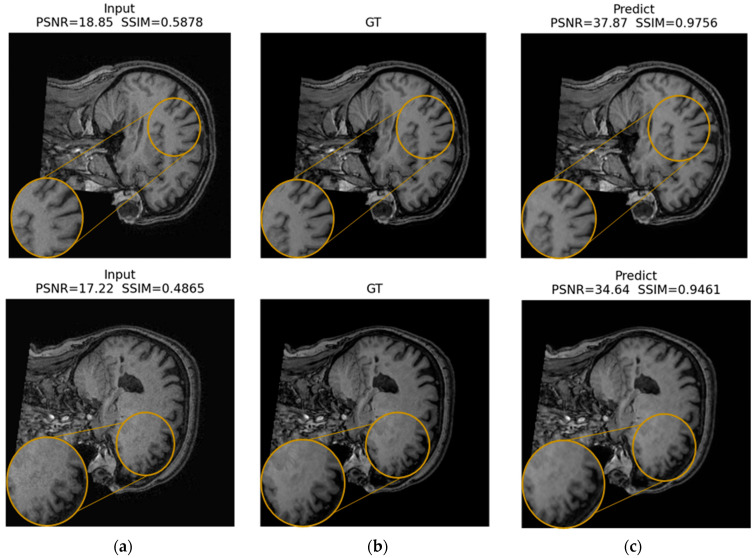
Visual comparison of zipper artifact correction. (**a**) Artifacted input image; (**b**) ground truth (GT); and (**c**) model prediction. Highlighted regions indicate areas affected by the zipper artifact, where the proposed model achieves noticeable improvements in image quality, reflected in higher PSNR and SSIM values.

Figure 16, Figure 17 and Figure 18 present qualitative examples of corrections for each artifact type, comparing the artifacted images, the ground truth, and the model’s predictions. In the ringing examples, per-edge oscillations at high-contrast transitions are consistently reduced, resulting in sharper edges at gray and white matter boundaries (Figure 16).

For herringbone, the high-frequency oblique bands are noticeably attenuated or disappear, while the fine anatomy of the cortex and deep structures is preserved, with textures and gray level distributions comparable to those of the ground truth (Figure 17).

Finally, in the zipper cases, the interference lines crossing the volume largely disappear in the predictions. This restoration produces a more homogeneous background and brain contours that are similar to those in the reference image, without an evident loss of contrast, and with improvements across all evaluation metrics (Figure 18).

The predictions closely resemble the artifact-free images, supporting the model’s ability to suppress distortions while preserving relevant structural information.

Across all three qualitative examples, the model consistently improves visual fidelity and reduces structured residuals in the absolute error maps |Input—GT| and |Pred—GT| (same z-score domain and shared color scale within each Figure). For ringing, the prediction restores sharper boundaries and suppresses oscillatory edge-related errors, increasing PSNR from 37.98 to 44.53 dB and SSIM from 0.9809 to 0.9956. For herringbone, the characteristic stripe-like pattern visible in the input error map is strongly attenuated after correction, with PSNR improving from 35.50 to 42.37 dB and SSIM from 0.9426 to 0.9901. For the more challenging zipper case, the input exhibits pronounced banding and large residuals, whereas the prediction substantially reduces the stripe energy and error magnitude, yielding a marked improvement in metrics (PSNR 17.26 to 29.63 dB, SSIM 0.4471 to 0.8681).

**Figure 19:**
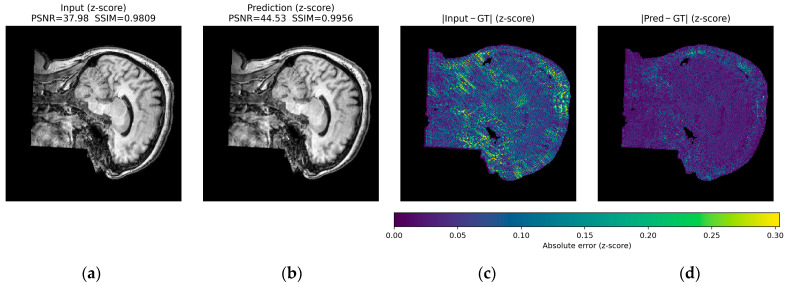
Ringing artifact suppression (z-score domain). (**a**) Input image with PSNR/SSIM, (**b**) model prediction, (**c**) absolute error map ∣Input—GT∣, and (**d**) absolute error map ∣Pred—GT∣. The prediction reduces edge-related ringing residuals (PSNR 37.98 → 44.53 dB, SSIM 0.9809 → 0.9956).

**Figure 20:**
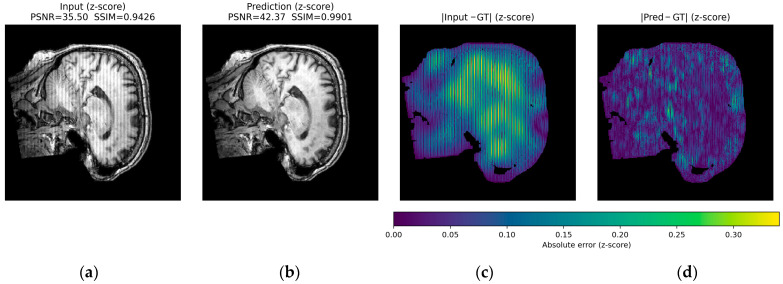
Herringbone artifact suppression (z-score domain). (**a**) Input image with PSNR/SSIM, (**b**) model prediction, (**c**) ∣Input—GT∣, and (**d**) ∣Pred—GT∣. The stripe-like residual pattern is markedly attenuated after correction (PSNR 35.50 → 42.37 dB, SSIM 0.9426 → 0.9901).

**Figure 21:**
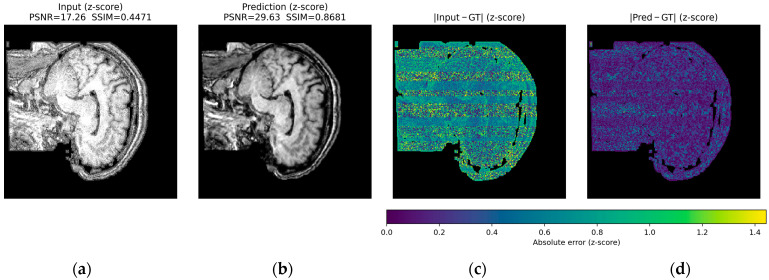
Zipper artifact suppression (z-score domain). (**a**) Input image with PSNR/SSIM, (**b**) model prediction, (**c**) ∣Input—GT∣, and (**d**) ∣Pred—GT∣. Strong banding artifacts and large residuals in the input are substantially reduced (PSNR 17.26 → 29.63 dB, SSIM 0.4471 → 0.8681).

In all cases, residual errors become more spatially diffuse and lower in magnitude after correction, indicating effective suppression of periodic artifact structure (Figure 19, Figure 20 and Figure 21).

**Figure 22:**
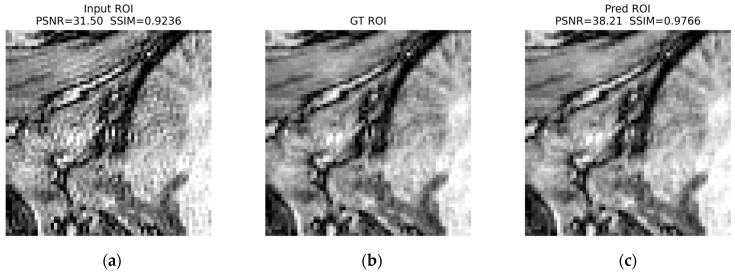
(**a**) Input ROI; (**b**) Ground truth (GT) ROI; (**c**) Predicted ROI. In the selected ROI, the prediction is closer to the ground truth and reduces ringing-related distortions. Quantitatively, PSNR increases from 31.50 dB to 38.21 dB (+6.71 dB), and SSIM increases from 0.9236 to 0.9766 (+0.0530).

In the ROI triptychs (a: Input ROI, b: GT ROI, and c: Pred ROI), the corrected ROIs show closer visual agreement with the ground truth images and reduced structured artifacts across all cases. For ringing (Figure 22), the prediction preserves fine anatomical edges while attenuating local oscillatory patterns: PSNR increases from 31.50 dB (input) to 38.21 dB (prediction), and SSIM increases from 0.9236 to 0.9766.

**Figure 23:**
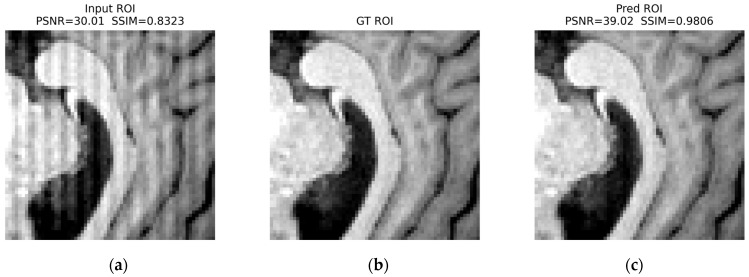
(**a**) Input ROI; (**b**) Ground truth (GT) ROI; (**c**) Predicted ROI. The ROI shows strong suppression of the stripe-like (herringbone) pattern in the prediction relative to the input, with improved similarity to the GT ROI. PSNR increases from 30.01 dB to 39.02 dB (+9.01 dB), and SSIM increases from 0.8323 to 0.9806 (+0.1483).

For herringbone (Figure 23), the stripe-like contamination visible in the input ROI is strongly reduced, producing a texture more consistent with the GT ROI: PSNR increases from 30.01 dB to 39.02 dB, and SSIM increases from 0.8323 to 0.9806.

**Figure 24:**
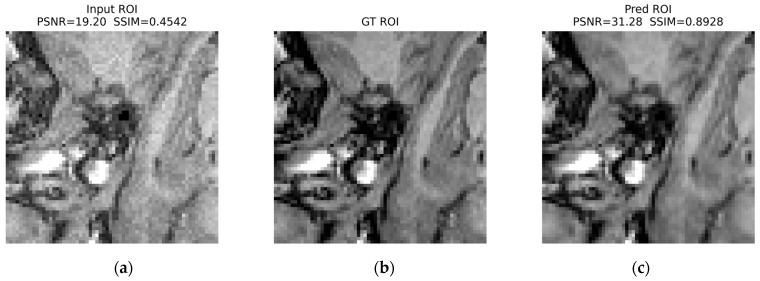
(**a**) Input ROI; (**b**) Ground truth (GT) ROI; (**c**) Predicted ROI. The prediction substantially reduces pronounced banding within the ROI and restores local contrast toward the GT ROI. PSNR increases from 19.20 dB to 31.28 dB (+12.08 dB), and SSIM increases from 0.4542 to 0.8928 (+0.4386).

For the zipper (Figure 24), the input ROI exhibits pronounced banding and contrast disruption; the prediction suppresses the band structure and restores local contrast toward the GT ROI: PSNR increases from 19.20 dB to 31.28 dB, and SSIM increases from 0.4542 to 0.8928.

### 4.7. Stress Test on Mixed Periodic Artifacts

To assess robustness within the controlled synthetic setting under combined degradations, we perform a stress test using synthetic mixtures of periodic artifacts generated by composing the same corruption operators used in the single-artifact setting. Specifically, we considered two-artifact mixtures (ringing+zipper, herringbone+ringing, and zipper+herringbone) and a three-artifact mixture (ringing+zipper+herringbone), where the degradations are applied sequentially to the GT to obtain a single mixed-input image.

Performance was evaluated using SSIM and PSNR, reported for Noisy(mix) vs. GT and Predict vs. GT. We also compute the paired gain per slice as Δ = Metric(Predict, GT) − (Noisy(mix), GT). Results are summarized as mean ± standard deviation, with gains reported as mean values (optionally with 95% bootstrap confidence intervals (CIs)).

Mixed artifacts substantially degrade image quality (low SSIM and PSNR), whereas the proposed model restores structural similarity and improves fidelity across all mixture conditions. This yields consistently positive ΔSSIM and ΔPSNR. Mean results for each mixture and for the stress test are reported in Table 10.

### 4.8. Results Stratified by Artifact Severity

Using the generator-defined severity parameters (intensity for ringing/zipper, smooth for herringbone) together with the p25/p75 thresholds (mild ≤ p25, severe ≥ p75, and moderate otherwise), we stratified the paired evaluation dataset (n = 12,529 slices). We then computed mean improvements in PSNR and SSIM in the z-score domain. Uncertainty was estimated using bootstrap 95% CI for the mean improvement (n_boot = 2000), and statistical significance was assessed with a paired sign-flip permutation test (n_perm = 10,000).

The model yields statistically significant improvement in the z-score domain. Across 12,529 paired samples, PSNR increases from 33.77 ± 11.62 dB at the input to 41.03 ± 6.15 dB at the output, corresponding to a mean gain of +7.26 dB (bootstrap 95% CI [7.15, 7.38]). Similarly, SSIM rises from 0.8323 ± 0.2190 to 0.9740 ± 0.0414, corresponding to a mean gain of +0.1418 (95% CI [0.1385, 0.1452]; paired sign-flip permutation *p* ≤ 1 × 10^−4^).

As shown in Table 11, the magnitude of improvement depends on both artifact type and severity. Zipper artifacts show the largest gains across all severity levels, with consistently strong improvements in both PSNR and SSIM. Ringing artifacts also improve significantly, although the gains are more moderate than those observed for zipper. For herringbone, the effect is severity-dependent: the mild group shows only a small improvement, whereas the moderate and severe groups exhibit clearly larger gains. The analysis of the results indicates that the proposed model is most effective when the degradation is stronger and more structurally pronounced, while still preserving stable performance across the full severity range.

### 4.9. Ablation Study on Radon-Based Loss Regularization

The ablation results show the contribution of the main components of the proposed architecture. The inclusion of the Radon-based regularization yields a gain of +0.686 dB in PSNR and +0.00286 in SSIM relative to the model trained without the Radon term, indicating that the Radon constraint improves the suppression of oriented periodic residuals.

Removing the attention mechanism results in greater degradation (−2.20 dB PSNR, −0.01233 SSIM), suggesting that attention plays an important role in selectively refining artifact-affected regions. The largest drop occurs when the AA component is removed (−8.46 dB in PSNR, −0.14313 in SSIM), indicating the importance of the low-frequency wavelet representation in preserving global anatomical structure. Removing the remaining wavelet sub-bands or their associated losses results in consistent degradation of roughly −5 dB in PSNR and −0.02 in SSIM, indicating that multi-sub-band supervision helps stabilize reconstruction across artifact patterns.

These results suggest that the combined components contribute jointly to the observed performance improvements. The ablation should still be interpreted as a component-level analysis rather than an assessment of each design choice. See Table 12.

### 4.10. Comparison of Results

This section presents and compares the results of all trained models with our proposed approach, which achieved the best performance (Table 13).

Based on the results presented in Table 13, the proposed model outperforms all trained baseline models, achieving the highest SSIM (0.98528 ± 0.02218) and PSNR (43.33710 ± 5.36451 dB). These results indicate a superior ability to preserve structural similarity and achieve higher reconstruction fidelity compared with the alternative approaches.

Furthermore, the proposed model exhibits one of the lowest standard deviations among all evaluated methods, suggesting greater stability and consistency across the test samples. The combination of high average metric values and low variability demonstrates the robustness of the proposed approach and increases confidence in its performance. In contrast, models such as Attention–Attention and Vision Transformer show a noticeable drop in performance, particularly in PSNR, indicating the limitations of purely attention-based mechanisms when trained with limited data or under strict reconstruction constraints.

We also report computational indicators for all evaluated models in Table 14 (NVIDIA RTX A6000, NVIDIA Corporation, Santa Clara, CA, USA; 300 W TDP). These include parameter count, approximate FLOPs/MACs for a fixed input size, peak GPU memory usage during training, average wall-clock training time per epoch, total training time normalized to 100 epochs, and inference latency per slice. All methods were profiled under the same wavelet-input regime (four db2 sub-bands) and comparable training settings to ensure a fair cost–performance comparison.

## 5. Discussion

Regarding the learning process, the composite loss decreases rapidly during the early epochs and then stabilizes within a low range for both training and validation, without sustained increases or marked divergence between the two curves. This behavior suggests that the model adapts its parameters to the data characteristics without clear evidence of severe overfitting, while maintaining stable performance on previously unseen volumes within the same controlled synthetic regime. The simultaneous stabilization of both curves indicates that the proposed architecture can capture the main structure of the problem without obvious signs of memorization in the training data. Furthermore, the SSIM and PSNR metrics increase across all three artifact types in the reported experiments.

Wavelet-domain analysis provides insight into how the model operates. The reduction in MSE after correction is particularly pronounced in the detail bands (AD, DA, and DD) for all three artifacts, whereas the approximation band (AA) changes to a lesser extent. This pattern suggests that most of the correction occurs in high-frequency components, where periodic structures and oscillations associated with ringing, herringbone, and zipper artifacts are concentrated, whereas changes in the low-frequency band appear comparatively smaller.

In the case of zipper artifacts, the reduction in error across all wavelet bands suggests a reduction in interference-line patterns, with no visually apparent loss of the underlying anatomical structures in the evaluated examples. However, this four-band representation also has limitations. Each band (AA, AD, DA, and DD) captures different frequency ranges and orientations (low-frequency, primarily horizontal, vertical, and diagonal details), resulting in a relatively rigid representation. Moreover, in the current architecture, the sub-bands are treated as independent channels, without explicitly modeling correlations across scales and orientations. This design may reduce sensitivity to more complex artifact patterns or anatomical structures that extend across multiple scales and orientations.

Although zipper artifacts yield the lowest absolute post-correction metrics (0.951 ± 0.023 in SSIM and 32.424 ± 2.427 dB in PSNR), compared with herringbone (0.990 ± 0.009 SSIM and 39.892 ± 3.046 dB PSNR) and ringing (0.995 ± 0.003 SSIM and 41.658 ± 2.062 dB PSNR), they also show the largest relative improvement in these metrics. The average SSIM increases by approximately 13 percentage points (from 0.860 to 0.951), whereas the improvement is about 2.2 points for herringbone (from 0.968 to 0.990) and about 1 point for ringing (from 0.987 to 0.995).

Although SSIM and PSNR increase substantially and visual inspection (Figure 16, Figure 17 and Figure 18) indicates marked artifact attenuation, the synthetic artifact generation process may not fully capture the variability and irregularity of real artifacts, particularly because no public datasets with annotated real MRI artifacts are currently available for direct validation.

In our setup, artifacts are generated through discrete, symmetric peaks in k-space, which introduces a structural bias into the training data. As a result, the training data are dominated by highly structured, regular, and periodic patterns, which can be interpreted as idealized artifacts. While this regular structure likely reduces variability in the learning problem, it may lead to an overestimation of the ability of the model to generalize to more complex, asymmetric, or irregular artifacts found in real clinical data.

Another important limitation concerns the Radon transform-based normalization used to align artifact orientation. As implemented, this procedure assumes a single dominant angle in the interference pattern and is therefore tailored to highly regular global structures. In practice, however, artifacts may exhibit multiple simultaneous orientations, local curvatures, or spatial variations. In such cases, estimating a single global angle through the Radon transform may average heterogeneous structures and reduce sensitivity to local components, thereby reducing correction performance for heterogeneous artifacts and biasing the model toward patterns that more closely match the synthetic design.

The proposed pipeline also introduces additional computational overhead compared with standard U-Net baselines. (i) Radon-guided angle normalization and its inverse rotation add pre- and post-processing steps and may introduce minor interpolation artifacts. (ii) DWT/IDWT operations and multi-band processing increase the computational footprint. (iii) Attention-gated skip connections increase parameterization and memory usage, and (iv) performance may also degrade when the dominant artifact orientation is ambiguous.

Nevertheless, the ablation study indicates that the Radon component has a measurable impact on the reported performance. Incorporating Radon-based regularization yields gains of +0.686 dB in PSNR and +0.00286 in SSIM, suggesting that orientation normalization is associated with improved correction performance in this synthetic setting.

The stratified evaluation shows that the model remains effective across different artifact severity levels, with the largest improvements observed in severe cases while still providing consistent benefits for milder degradations. Similar trends are reflected in the absolute error maps, which visually indicate marked attenuation of artifact-related distortions, and in the ROI-based analysis, where improvements are also observed in anatomically relevant regions. These findings suggest that, in this controlled synthetic setting, the method improves global similarity metrics and local image quality in structurally relevant regions.

The mixed-artifact experiments provide additional insight into the model’s behavior under more challenging conditions. Although the network was trained on individual artifact types, it still improves on inputs altered by unseen synthetic combinations of the same periodic corruption operators, suggesting limited generalization in this synthetic setting.

Finally, the edge-preservation analysis—based on Sobel-derived representations and complementary metrics such as edge-PSNR, edge-SSIM, Grad-L1, Grad-L2, Edge Dice/F1, and the Radon peak ratio—indicates that, within this synthetic evaluation, increases in the considered metrics are not accompanied by obvious losses of structural information. Instead, the results suggest that, in this controlled synthetic setting, the proposed approach suppresses periodic artifacts while preserving significant anatomical boundaries relevant for MRI post-processing analyses.

Another aspect to consider is extending the problem to 3D and the inter-slice coherence. The current model operates on independent 2D slices and therefore does not explicitly exploit spatial correlations along the slice axis, where many artifacts—particularly those related to hardware or motion—manifest continuously across slices. In addition, uncertainty remains regarding the severity ranges used during synthetic artifact generation (peak amplitude in k-space, number of spikes, noise levels, etc.). If these parameters are tuned toward relatively mild conditions, the model may be better adapted to correcting subtle artifacts but less robust to more extreme cases.

Finally, although the inverse Radon and wavelet transforms are theoretically well-defined and approximately reversible, their practical implementation involves interpolation, boundary effects, and numerical quantization. These steps may introduce local smoothing, slight geometric misalignments, or small redistributions of high-frequency energy.

Although such effects are typically small, they are not entirely negligible and should be considered when interpreting the corrected images as approximations to the original anatomy, especially when several transforms are applied sequentially within the same processing pipeline.

## 6. Conclusions

In the controlled synthetic evaluation, the model increases image similarity metrics, with higher SSIM and PSNR values. It also attenuates synthetically generated ringing, herringbone, and zipper artifacts, with the largest relative gains observed for zipper artifacts, accompanied by a marked reduction in wavelet-domain error, particularly in the detail bands.

This approach also presents several limitations. Synthetic artifacts arise from discrete, symmetric peaks in k-space and are represented using four wavelet sub-bands that are processed largely independently. This configuration favors learning regular, globally oriented patterns and does not guarantee robustness to more complex, irregular, or locally heterogeneous artifacts observed in real data. In addition, the use of the Radon transform to normalize orientation assumes a single dominant angle, the model operates on 2D patches without explicitly enforcing inter-slice coherence, and the inverse Radon and wavelet transforms may introduce interpolation and boundary effects. These aspects can lead to biases and small distortions that may limit generalization to real-world clinical settings.

Within these constraints, the reported results provide a methodological validation of periodic artifact correction in a controlled synthetic environment. They also underline the need to generate more realistic artifact models, explore architectures operating directly in 3D, and develop formulations that explicitly exploit dependencies across scales, orientations, and slices.

## Figures and Tables

**Table 1:** Summary of deep learning-based approaches for artifact correction in medical imaging, including artifact category, type, principal causes, solution approaches, and key limitations.

Category	Field	Content
Sampling, aliasing and truncation	Artifacts	Aliasing, Gibbs, ringing, Wrap-around, reduced spatial resolution
	Main causes	Signal discretization in space and frequency, Undersampling, k-space truncation, Signal outside the field of view
	Deep learning solution approaches	CNN/ResNet/autoencoder reconstruction [1,2,3,4,5,9,10,11,12,13,14,15]; zero-shot and in situ adaptation [6,7]; adversarial and dual-domain learning [8]; structured k-space correction [16,17]; model-based unrolling [22,23,24]; transformer reconstruction [31,32,33,34,35]; hybrid pipelines [8,22,23,24,31,32,33,34,35]
	Identified limitations	Architectural complexity in dual-domain models [8]; limited 3D and low-SNR validation [6,7]; high computational cost [10]; possible suppression of subtle findings [11]; limited pathological validation [12,13,14]; metric shifts under threshold-based evaluation [15]; dependence on training data [16]; latency in iterative methods [36,37,38,39]
Motion artifacts	Artifacts	Blurring, ghosting, phase stiffness, respiratory artifacts, motion shifts in DWI
	Main causes	Voluntary and involuntary patient motion, breathing, fetal motion, cardiac motion
	Deep learning solution approaches	CNN/U-Net restoration [41,42,43,44,51,52]; GAN-based approaches [45,48,55]; diffusion and score-based models [46,47,54]; unpaired and autoencoder pipelines [50,56]; hybrid motion estimation + restoration [41,42,43,44,45,46,47,50,51,52,53,54,55,56]
	Identified limitations	GAN instability and possible anatomical artifacts [45]; high computational cost in diffusion models [46,47]; limited multicenter and real-time 3D/4D validation [53]; sensitivity to hyperparameters and limited pathological evaluation [55,56]
Off resonance and susceptibility B0	Artifacts	Geometric distortions, signal voids from metals, signal mismatches, frequency shifts
	Main causes	B0 field inhomogeneity, susceptibility differences between tissues, metallic implants, and incomplete shimming
	Deep learning solution approaches	ΔB0-based geometric correction [63,64,65]; CNN/U-Net restoration [3,58,61,62]; unsupervised and multimodal approaches (dual polarity, CT–MRI transfer, GANs) [57,60,62]; hybrid field-map + restoration pipelines [57,58,59,60,61,62,63,64,65]
	Identified limitations	Additional acquisitions may increase scan time [57]; dependence on reversed polarity data [63]; possible alteration of derived metrics (e.g., FA in TBSS) [62]; training sensitivity to hyperparameters [65]; robustness to motion and protocol variability remains limited [63]
Ghosting phase, errors and system effects	Artifacts	Herringbone spike and corduroy patterns, zipper artifacts, ghosting from flow and pulsatility, gradient nonlinearities, eddy currents
	Main causes	Phase errors, RF interference and unintended RF emissions, system nonlinearities and gradient faults, eddy currents, flow, and pulsatility
	Deep learning solution approaches	CNN/U-Net restoration [66,68]; coil-combination and subspace methods [63,67]; generative and hybrid approaches for metal/flow artifacts [61,69]; physics-informed pipelines with CNN post-processing [63,66,67,68,69]
	Identified limitations	Dependence on physical model assumptions [66]; computational cost at high resolution [66]; limited generalization with task-specific annotations [61]; robustness across scanners and protocols still under evaluation [67]

**Table 2:** Parameters for controlling the severity of the ringing artifact.

Parameter	Lower Limit Value	Higher Limit Value	Type
Scalar intensity (α)	0	1	Decimal
Propagation axis	0	2	Integer
End angle ($\theta_{2}$)	0	360	Integer
Initial angle ($\theta_{1}$)	$\theta_{2}-100$	$\theta_{2}-10$	Integer
Outer radius ($r_{e}$)	118	123	Integer
Inner radius ($r_{i}$)	$r_{e}-20$	$r_{e}-10$	Integer

**Table 3:** Parameters controlling the severity of the herringbone artifact.

Parameter	Lower Limit Value	Higher Limit Value	Type
Smoothing $\left(S\right)$	3	20	Integer
Propagation axis	0	2	Integer
Selection point	0	3	Integer
Kernel size ($k_{S}$)	3	13	Integer
Distance $\left(d\right)$	−30	30	Integer

**Table 4:** Parameters for controlling the severity of zipper artifacts.

Parameter	Lower Limit Value	Higher Limit Value	Type
Intensity	15	50	Integer
Propagation axis	0	2	Integer
Number of artifacts	1	16	Integer
Variability	20	40	Integer
Amplitude	10	50	Integer

**Table 5:** Hyperparameter values.

Parameter	Value
Input/Output Bands	4 (AA, AD, DA, DD)
Encoder/Decoder Levels	4/4
Loss	WaveletLoss (band-weighted L1)
Metrics	SSIM and PSNR (image), MAE per band
Optimizer	Adam
Batch Size/Epochs	8/50
Dataloader Workers	4
Wavelet	db2
Minimum Tile Size	≥32 × 32

**Table 6:** Results of multivariate analysis on wavelet + GLCM characteristics in training and validation.

Test	Stat	*p*-Value
Energy Distance	0.010375	<0.005
MMD-RBF	0.000320	<0.005
Sliced-Wasserstein	0.073419	<0.005

**Table 7:** Results of the wavelet family selection experiment (db1–db6) for brain MRI artifact correction using a U-Net architecture.

Wavelet Family	SSIM	MAE
db2	0.97322 ± 0.00217	0.03570 ± 0.00051
db4	0.97306 ± 0.00203	0.03535 ± 0.00046
db3	0.97180 ± 0.00139	0.03552 ± 0.00085
db1	0.97149 ± 0.00252	0.03721 ± 0.00119
db6	0.97068 ± 0.00219	0.03541 ± 0.00101
db5	0.97049 ± 0.00244	0.03641 ± 0.00134

**Table 8:** Average MSE per wavelet component and overall SSIM and PSNR across the entire image.

Metric	Art Value	Correction Value
MSE_$w_{AA}$	0.048 ± 0.869	0.007 ± 0.009
MSE_$w_{AD}\;$	0.008 ± 0.011	0.003 ± 0.004
MSE_$w_{DA}$	0.009 ± 0.011	0.004 ± 0.004
MSE_$w_{DD}$	0.007 ± 0.010	0.002 ± 0.003
MSE_$w_{mean}$	0.018 ± 0.029	0.004 ± 0.005
PSNR [dB]	33.42 ± 6.939	43.337 ± 5.364
SSIM	0.938 ± 0.067	0.985 ± 0.022

**Table 9:** Quantitative evaluation of artifact correction and edge preservation (input vs. output).

Metric	$\left|Input–GT\right|$	$\left|Output–GT\right|$
Edge-PSNR	38.41 ± 12.39	41.04 ± 8.54
Edge-SSIM	0.952 ± 0.121	0.980 ± 0.060
Grad-L1	0.0744 ± 0.0530	0.0540 ± 0.0392
Grad-L2	0.0622 ± 0.0466	0.0436 ± 0.0342
Edge Dice/F1	0.920 ± 0.0500	0.938 ± 0.0416
Radon peak ratio	6.72 ± 10.33	2.89 ± 3.46

**Table 10:** Stress test on mixed periodic artifacts.

Mixture Artifact	n	PSNR in	PSNR out	SSIM in	SSIM out	ΔPSNR	ΔSSIM
Ringing + zipper	150	17.83 ± 3.00	30.61 ± 3.39	0.4621 ± 0.1336	0.8911 ± 0.0767	+12.78	+0.4290
Herringbone + ringing	150	35.13 ± 2.86	38.54 ± 2.84	0.9600 ± 0.0208	0.9793 ± 0.0144	+3.41	+0.0192
Zipper + herringbone	150	17.64 ± 3.21	29.07 ± 2.88	0.3902 ± 0.1557	0.8579 ± 0.0668	+11.43	+0.4678
Ringing + zipper + herringbone	150	17.58 ± 3.35	28.95 ± 2.87	0.3950 ± 0.1582	0.8570 ± 0.0701	+11.37	+0.4620

**Table 11:** PSNR and SSIM results stratified by artifact severity (input vs. output) by artifact type.

Artifact	Severity	Severity Interval	n	PSNR in	PSNR out	SSIM in	SSIM out
herringbone	mild	smooth ≤ 6	573	47.13 ± 2.88	47.34 ± 1.65	0.9948 ± 0.0031	0.9966 ± 0.0017
herringbone	moderate	6 < smooth < 16	2414	40.88 ± 3.60	44.35 ± 2.68	0.9790 ± 0.0132	0.9930 ± 0.0069
herringbone	severe	smooth ≥ 16	903	37.41 ± 3.44	42.63 ± 2.60	0.9618 ± 0.0176	0.9899 ± 0.0096
ringing	mild	intensity ≤ 0.27195	1386	42.57 ± 5.59	45.50 ± 3.91	0.9886 ± 0.0121	0.9950 ± 0.0048
ringing	moderate	0.27195 < intensity < 0.76809	2038	42.39 ± 5.11	44.93 ± 3.58	0.9896 ± 0.0093	0.9950 ± 0.0037
ringing	severe	intensity ≥ 0.76809	982	40.17 ± 4.56	44.24 ± 3.53	0.9854 ± 0.0104	0.9945 ± 0.0040
zipper	mild	intensity ≤ 22	658	20.90 ± 1.37	35.77 ± 3.59	0.6531 ± 0.0468	0.9537 ± 0.0321
zipper	moderate	22 < intensity < 41	2579	18.49 ± 1.83	34.08 ± 3.02	0.5267 ± 0.0855	0.9384 ± 0.0410
zipper	severe	intensity ≥ 41	996	17.41 ± 2.03	32.04 ± 3.72	0.4848 ± 0.0858	0.9142 ± 0.0757

**Table 12:** Quantitative ablation study of the main architecture components.

Model	SSIM	PSNR
Our model	0.98528 ± 0.02218	43.33710 ± 5.36451
without Radon	0.98242 ± 0.02441	42.65113 ± 5.73252
without Attention	0.97295 ± 0.04182	41.13236 ± 6.58765
without AA component	0.84215 ± 0.08133	34.87268 ± 7.15305
without AD component	0.95828 ± 0.04865	37.92928 ± 5.17067
without DA component	0.96147 ± 0.04702	38.48882 ± 5.56199
without DD component	0.95989 ± 0.05004	38.30767 ± 5.53929
without AA loss	0.96311 ± 0.04702	38.36318 ± 5.26245
without AD loss	0.96266 ± 0.04576	38.05932 ± 4.99527
without DA loss	0.96354 ± 0.04467	38.21393 ± 5.06365
without DD loss	0.96377 ± 0.04325	38.17828 ± 4.85161

**Table 13:** Average SSIM and PSNR results for each trained model compared with the proposed approach.

Model	SSIM	PSNR
Our model	0.98528 ± 0.02218	43.33710 ± 5.36451
U-Net	0.97295 ± 0.04182	41.13236 ± 6.58765
GAN	0.97390 ± 0.03990	41.08036 ± 6.32473
Spatial + channel attention	0.97353 ± 0.03422	41.41551 ± 6.70846
Attention–attention	0.93782 ± 0.04089	32.84799 ± 3.70444
Vision transformer	0.97322 ± 0.04279	40.77219 ± 6.18026

**Table 14:** Computational cost comparison across the different approaches.

Model	Params (M)	FLOPs/ MACs (G)	Peak GPU Mem (GB)	Train Time /Epoch (s)	Total Train Time (min, 100 ep)	Inference Latency/Slice (ms)
Our model	33.482	14.337	1.433 ± 0.03	359.83 ± 10.79	599.72 ±17.99	5.461 ± 0.27
U-Net	31.391	13.933	1.582 ± 0.03	335.07 ± 10.05	558.45 ± 16.75	4.399 ± 0.22
GAN	34.155	13.933	1.583 ± 0.04	671.05 ± 26.84	1118.42 ± 44.74	4.389 ± 0.22
Spatial + channel attention	32.177	14.451	4.554 ± 0.09	601.78 ± 24.07	1002.97 ± 40.12	6.202 ± 0.37
Attention–attention	3.599	9.881	4.528 ± 0.09	4324.75 ± 216.24	7207.92 ± 360.4	23.526 ± 1.41
Vision transformer	43.508	14.442	1.968 ± 0.04	529.47 ± 21.18	882.44 ± 35.30	12.327 ± 0.74

## Data Availability

The data presented in this study are openly available in Dataset Creation: Synthetic MRI Artifacts (Ringing, Herringbone, Zipper) at https://github.com/Jesusdrp09/Periodic-Artifact-Dataset-Creation-in-Brain-MRI (accessed on 20 February 2026).

## Abbreviations

The following abbreviations are used in this manuscript:

AA	Approximation–approximation
AD	Approximation–detail
DA	Detail–approximation
DD	Detail–detail
DWT	Discrete wavelet transform
IDWT	Inverse discrete wavelet transform
